# Factors affecting veterinary perception of the prevalence of back pain and dysfunction in dogs and horses

**DOI:** 10.3389/fvets.2026.1857696

**Published:** 2026-08-17

**Authors:** David Lane, Kevin K. Haussler

**Affiliations:** 1 Points East West Veterinary Services, Squamish, BC, Canada; 2 College of Veterinary Medicine, Lincoln Memorial University, Harrogate, TN, United States

**Keywords:** back examination, back pain and dysfunction, canine back pain, equine back pain, myofascial trigger point, nonspecific back pain, vertebral motion segment

## Abstract

**Introduction:**

The prevalence of back pain in humans has been extensively reported, but in dogs it is unknown. In horses, research on the prevalence of back pain exists, but with reports ranging from 1% to 94%, indicating a data deficiency.

**Methods:**

Veterinarians were surveyed about their perception of the prevalence of back pain and dysfunction in dogs and horses for 6 different clinical scenarios. The respondents’ education background and physical examination techniques were recorded.

**Results and discussion:**

Perceptions of the prevalence of back pain and dysfunction varied widely both within and between education groups. Sports medicine and rehabilitation specialists, and veterinarians certified in either spinal manipulation therapy or acupuncture, were more likely to report the presence of back pain and dysfunction compared to other practitioners. Clinicians that examined individual vertebral motion segments, or palpated for myofascial trigger points, appreciated a greater prevalence of back pain and dysfunction than those who did not. Respondents’ perception is that primary axial issues are a common cause of musculoskeletal pain, and can clinically present as unilateral limb lameness. Compared to appendicular lesions, primary axial lesions are more likely to be found in patients presented with a non-specific reduced athletic performance. Respondents report frequently detecting axial lesions in patients presented with no clinical history of pain, and as an additional finding in patients with a known primary limb lameness.The above findings are derived from the subjective opinion of self reporting respondents and do not necessarily represent true prevalence of back pain and dysfunction; further quantitative research using objective methods is required to make that determination. These results do underscore the need for improved veterinary education on how to assess back pain, myofascial tone, and spinal range of motion. Greater agreement between clinicians might also be achieved by developing a standardized reporting method that semi-quantifies the severity of pain and dysfunction individually. Additionally, standardizing and reporting spinal physical examination methodology may also facilitate consistent communication of clinical findings and greater agreement between clinicians.

## Introduction

In humans, low back pain has a lifetime prevalence of 84% and is the second most common complaint reported to general practitioners (1, 2). The sacroiliac joint is thought to be the primary source of discomfort in 15 to 30% of low back pain patients (3–5). Dysfunction originating from the costovertebral and costotransverse joints are also recognized as a source of thoracic and shoulder pain in humans (6, 7). Ninety percent of chronic low back pain is considered ‘non-specific’, meaning that no causal lesion can be identified based on medical history, physical examination, diagnostic testing, or imaging (1, 8). Clinical signs often include pain, impaired movement, muscle guarding, altered posture, reduced motor control, and muscle atrophy (4, 9). Whether the above information can be applied to veterinary patients is largely unknown.

In horses, back pain and dysfunction (BPAD) is considered a common cause of reduced athletic performance and behavioral issues (10, 11). The prevalence of BPAD reported in the equine literature ranges from 1 to 94% (11, 12). Impinged spinous processes, articular process osteoarthritis, epaxial muscle soreness, lumbosacral intervertebral disk degeneration, and sacroiliac joint osteoarthritis are all documented causes of equine back pain (10, 13, 14). Appendicular lameness has also been associated with cervical and cranial rib lesions, as well as thoracolumbar and pelvic pain, (10, 15–19). However, variable degrees of spinal pathologic findings have also been reported in presumed clinically normal horses (11, 16, 20). Conversely, horses with a history of reduced athletic performance and palpable back pain may have no evident spinal pathology on imaging, consistent with non-specific back pain in humans (11). An additional complication is that BPAD in horses is often considered secondary to limb lameness and is therefore frequently deemed clinically insignificant (21–23).

In dogs, back pain and neurologic deficits attributable to conditions such as intervertebral disk disease, degenerative lumbosacral stenosis, and discospondylitis are well documented (24), and spinal or back related musculoskeletal disease was the reason for 27% police dogs and 16% of military working dogs to be removed from duty (25, 74). While thoracolumbar and sacroiliac joint osteoarthritis occurs frequently in dogs, these disease processes are not recognized sources of back pain, despite being considered as such in both horses and humans (3, 4, 8, 11, 26–29). Except for a small case series, costal pathology has not been reported to contribute to forelimb lameness in dogs, as it has in horses (30). Back pain without demonstratable spinal lesions (i.e., non-specific back pain) has not been reported in dogs (31).

Orthopedic examination of appendicular structures is well described and considered a critical component of identifying the presence and location of limb lameness in veterinary medicine, but the same cannot be said for the examination of the axial region. Examination recommendations vary widely from no mention of spinal evaluation techniques in relevant textbooks, to a comprehensive assessment of active and passive spinal motion in static and dynamic conditions with methodical soft tissue palpation, and detailed assessment of vertebral segment pain, stiffness, and muscle hypertonicity (13, 32, 39, 75). Published data about the clinical significance of axial examination findings is scarce.

Anecdotally, many traditional veterinary education programs do not include training in physical examination techniques of axial structures. This deficiency likely contributes to reduced awareness of, and ability to, diagnose BPAD in veterinary patients. An increased interest in the presence and perceived high prevalence of neck and back pain in horses has driven recent advancements in anatomical, biomechanical, pathologic, and diagnostic imaging studies, as well as advancements in medical and surgical management options (21, 33, 34). The increased awareness of, and literature addressing axial issues in horses has seemingly outpaced that reported in small animal practice. Therefore, there may be a much greater awareness of BPAD among equine practitioners relative to canine practitioners.

A vertebral motion segment consists of two contiguous vertebrae and is considered the functional unit of the spine (13, 35). The vertebral column’s flexibility results from multiple vertebral motion segments working together, each contributing a small amount to overall spinal mobility (4, 13, 35). In human patients, detailed spinal evaluation routinely involves assessment of each vertebral motion segment for an abnormal neutral position, restricted joint range of motion, altered joint end feel, and localized pain (36–38). Motion segment dysfunction occurs with soft tissue pathology, which is palpably manifested as segmental hypomobility or hypermobility, or as the asymmetric neutral position of an individual vertebra (39–41). Pain is not a prerequisite for the presence of spinal dysfunction. Although the above examination techniques are routinely performed, conflicting data have been reported regarding the reliability of spinal palpatory tests in humans, with little veterinary data available, particularly for dogs (42, 43).

Examination of axial myofascial structures includes an assessment of muscle mass, tone, and pain (4, 9, 28). Muscle pain (myalgia) can be due to many causes, including myofascial trigger points which are characterized as palpable taut bands of hyperirritable muscle (44). Myofascial trigger points are simultaneously thought to be both a common but underdiagnosed cause of muscular pain in human and veterinary patients (44–48). Forty-two percent of working police dogs and 54% of dogs with osteoarthritis were found to have thoracolumbar epaxial myofascial trigger points (45, 46). Myofascial trigger points have been identified in horses as well, and may be associated with compensatory changes associated with chronic limb lameness and girth-aversion pain behaviors (49). Despite such findings, there is ongoing debate regarding the definition, diagnostic reproducibility and clinical significance of myofascial trigger points (50, 51).

Spinal evaluation techniques used by human physiotherapy, chiropractic, and acupuncture professionals have been successfully applied to veterinary patients (22, 39). Post graduate certification programs train veterinarians how to perform detailed axial physical examination. Spinal manipulation (chiropractic) programs focus on teaching vertebral motion segment examination techniques as described above. Acupuncture certification courses teach myofascial examination techniques and detection of myofascial trigger points. Canine rehabilitation therapy courses (Certified Canine Rehabilitation Therapist [CCRT] and Certified Canine Rehabilitation Practitioner [CCRP]) also teach the concepts of joint end feel, AROM, and PROM. It is unknown if traditional veterinary education programs provide formal training in these types of focused spinal evaluation techniques.

It is unclear why the veterinary profession has not adopted the term ‘non-specific back pain’. Possible explanations are that non-specific back pain does not exist in veterinary patients; that a translation of disease incidence between bipedal humans and quadrupedal canine or equine patients cannot be made. Perhaps non-specific back pain exists in dogs and horses, but clinicians presume that it is always secondary to overt spinal pathology. Perhaps it exists but is under diagnosed because veterinarians are not routinely performing the detailed spinal evaluation techniques employed by human medical professionals. Although objective determination of the prevalence of BPAD is ideal, an initial step toward clarifying the above is to first determine veterinarians’ perception of the prevalence of BPAD and how that perception varies in relation to education background, species being assessed (canine vs. equine), and the spinal examination techniques employed. To that end, an online survey was created to collect information about canine and equine practitioners’ perspectives on BPAD in their patients.

It is expected that opinions on the presence and prevalence of BPAD varies widely among veterinarians. Specifically, we hypothesize that: (1) veterinarians with different education backgrounds will report a different prevalence of BPAD, such that individuals with additional training (board certification in sports medicine and rehabilitation, or certification in spinal manipulation, acupuncture, or canine rehabilitation) are more likely to identify patients with BPAD compared to practitioners without these qualifications, (2) equine veterinarians will identify BPAD in a higher proportion of their patients compared to canine veterinarians, and (3) that practitioners who perform assessment of individual vertebral motion segments or palpate for myofascial trigger points will report a higher prevalence of BPAD compared to those who do not.

## Methods

### Initial survey development

An online survey was created to capture information on practitioner demographics, their perception of the prevalence of BPAD across six different clinical presentations (i.e., scenarios), and methods used for spinal examination. During the month of December 2024, an initial draft of the survey was distributed to 12 veterinarians, including eight general practitioners, three of which had additional certification in veterinary spinal manipulation, acupuncture, or rehabilitation therapy. One of the 12 respondents had an equine exclusive practice, one treated both dogs and horses, and the remainder treated dogs exclusively. Two respondents were boarded surgeons (American College of Veterinary Surgeons), one was boarded in sports medicine and rehabilitation (American College of Veterinary Sports Medicine and Rehabilitation), and one was boarded in neurology (American College of Veterinary Internal Medicine - Neurology). Access to the survey was provided via an online platform.^1^ Comments and suggested edits were considered and incorporated, as indicated, into the final survey (Supplementary Data Sheet 3).

### Survey design and distribution

The final survey consisted of three sections. In the first section, respondents were asked to report on their education background, whether they worked primarily with dogs, horses, or both, and if they were employed in an academic or non-academic (i.e., private practice) setting. Respondents that reported working in a combination of academia and private practice were categorized as working in academia. Respondents were then asked about their training in axial and appendicular examination techniques as part of their formal veterinary education. Educational background was assessed by recording relevant certifications and board specializations (Table 1). Both canine and equine respondents were categorized based on education background, divided into those with a veterinary degree but no additional certifications or board specializations (DVM), those with board specialization, and those with certification in acupuncture (CVA), or spinal manipulation therapy (CSMT). Canine respondents were afforded an additional category (CCR) for those with certification in canine rehabilitation therapy. Respondents with more than one certification were categorized as described in Table 1.

**Table 1:** Educational qualifications of respondents.

Educational qualifications	Abbreviation	Description
General practitioner	DVM	Graduate veterinarians with no additional specialty board status or certification in spinal manipulation therapy, acupuncture, or canine rehabilitation.
Board specialty in veterinary surgery (ACVS, ECVS)	VS	Graduate veterinarians with boarded surgical specialist status. May include additional certification in spinal manipulation therapy, acupuncture, or canine rehabilitation, but does not include boarded specialty status in sports medicine and rehabilitation.
Board specialty in veterinary sports medicine and rehabilitation (ACVSMR, ECVSMR)	VSMR	Graduate veterinarians with board specialization in sports medicine and rehabilitation. May include additional certification in spinal manipulation therapy, acupuncture, or canine rehabilitation, or boarded specialty status.
Other boarded veterinary specialists	VSP	Graduate veterinarians with boarded specialization in a field besides surgery, or sports medicine and rehabilitation. May include additional certification in spinal manipulation therapy, acupuncture, or canine rehabilitation.
Certified in canine rehabilitation	CCR	Graduate veterinarians with certification in canine rehabilitation, but no boarded specialty status or certification in acupuncture or spinal manipulation therapy.
Certified in canine or equine acupuncture	CVA	Graduate veterinarians with certification in acupuncture, but no boarded specialty status or certification in spinal manipulation therapy, but may have certification in canine rehabilitation.
Certified in canine or equine spinal manipulation therapy	CSMT	Graduate veterinarians with certification in spinal manipulation therapy, but no boarded specialty status. May have certification in acupuncture or canine rehabilitation.

Boarded specialists were divided into three categories: surgeons (VS), sports medicine and rehabilitation (VSMR), or boarded specialization in a field other than surgery or sports medicine (VSP). Individuals dual-boarded in sports medicine and surgery were assigned to the VSMR category. Respondents in the VSMR, CCR, CVA, and CSMT categories were designated as having additional training in axial examination techniques. All others (DVM, VS, and VSP) were considered to have no additional training in assessing axial issues.

In the second section of the survey, respondents were directed to either a canine or equine specific series of questions that included six different clinical scenarios aimed at determining the perceived prevalence of axial issues (Table 2). Mixed animal practitioners were asked to complete both the canine and equine sections. Scenario 1 included patients presented with any form of musculoskeletal pain, lameness, or dysfunction. Scenario 2 described patients with a unilateral limb lameness as the primary presenting complaint. Scenario 3 included two subgroups (3a dogs, 3b horses) with species-specific clinical signs indicative of primary BPAD. Scenario 4 represented patients with reduced athletic performance but no other indicators of musculoskeletal pain or lameness. Scenario 5 represented patients with no medical history indicating the presence of musculoskeletal pain or dysfunction. For each of these scenarios, respondents were asked to state the likelihood that the patient has (1) a primary axial lesion, (2) a primary appendicular lesion, and (3) concurrent axial and appendicular lesions that confound the ability to determine which is the primary or inciting cause. Scenario 6 asked what percentage of patients presented with a primary appendicular lameness also had secondary or concurrent BPAD.

**Table 2:** Clinical presentations and justification for inclusion in the survey.

Scenario	Clinical presentation	Justification
1	Prior diagnosis of musculoskeletal pain, lameness, or dysfunction	To determine the prevalence of primary axial versus appendicular disorders among patients with musculoskeletal pathology
2	Unilateral limb lameness	To determine the percentage of unilateral limb lameness attributable to a primary axial musculoskeletal disorder
3a	Canine: Altered spinal posture, pain when turning or bending, or reluctance to move	To determine the percentage of primary axial musculoskeletal disorders in patients with a clinical history strongly suggestive of BPAD
3b	Equine: Altered spinal posture, bucking, or adverse behavior with saddle placement	
4	Non-specific reduced athletic performance but no lameness (e.g., reduced jump height or speed)	To determine if canine patients with axial musculoskeletal disorders have decreased athletic performance
5	No known signs of musculoskeletal pain, dysfunction, or reduced athletic performance	To determine the frequency that axial musculoskeletal disorders are found on physical examination in patients with no history of musculoskeletal pain or dysfunction
6	Primary appendicular lameness	To determine the frequency that BPAD is detected in patients with a primary appendicular musculoskeletal lameness

The third section of the survey asked respondents for details on how they perform an axial examination. Specifically, if they assessed the vertebral column as a whole, by region (i.e., cervical, thoracic, lumbar, pelvis), or by individual vertebral motion segment. Respondents were also asked if they examined the sacroiliac joints or ribs. Those that reported examining individual vertebral motion segments, sacroiliac joints, or ribs were then asked whether they assessed pain, AROM, PROM, neutral position, joint end-feel, or “other – fill in the blank.” Respondents were also asked about their evaluation of the paraspinal musculature, specifically whether they palpate for myalgia, muscle tone, myofascial trigger points, muscle mass, or “other – fill in the blank.”

The survey was uploaded to an online survey platform (see Footnote 1) and distributed through private discussion groups for veterinary neurology, surgery, and sports medicine specialists. It was added to newsletters for both veterinary surgery and sports medicine specialists, and for rehabilitation certified general practitioners. It was also posted on multiple private veterinary Facebook groups (Table 3). The survey was open for responses for two months (from January 14, 2025 to March 16, 2025). Respondents needed to answer at least one question from sections 2 or 3 to be included in the results. Respondents that provided information on their education background and what species they treat, but did not answer questions regarding the different clinical scenarios and examination techniques, were excluded. To be included, individuals needed to report possessing a doctoral degree in veterinary medicine (DVM or equivalent) without any consideration about the nature of their practice (research vs. clinical practice, retired vs. actively employed, etc.).

**Table 3:** Facebook groups, private discussion groups, and newsletters to which the survey was distributed.

Facebook organization	URL
49th North Veterinarians	https://www.facebook.com/groups/1097394794085917
Canadian Animal Rehab Support	https://www.facebook.com/groups/752787724869613
Small Animal Surgery Discussion Page	https://www.facebook.com/groups/smallanimalsurgery.org
Veterinary ECC Small Talk	https://www.facebook.com/groups/321900661313541
Small Animal Medicine Discussion Page	https://www.facebook.com/groups/295725880776912
Veterinary Network	https://www.facebook.com/groups/371096630961250
Veterinarian Palliative Medicine Group	https://www.facebook.com/groups/worldvetpalliativemedicine
Vet Med RAW	https://www.facebook.com/groups/2290080361203545
Small Animal Rehabbers	https://www.facebook.com/groups/976440089198714
Veterinary Woman Group	https://www.facebook.com/groups/463161975066166
Veterinary Orthotics and Prosthetics	https://www.facebook.com/groups/560598999570355
Veterinary Manual Therapy	https://www.facebook.com/groups/1862218003961604
Vets Without Kids	https://www.facebook.com/groups/1783218795224556
Holistic Veterinarian-Only Discussion Group	https://www.facebook.com/groups/372570869797326
Australian Veterinarian Network	https://www.facebook.com/groups/Australianvetnetwork
DVM Equestrians	https://www.facebook.com/groups/707213993423175
ACVS Diplomates	https://www.facebook.com/groups/125304600661
Vet2Vet	https://www.facebook.com/groups/336298203233101
For Veterinary Use Only	https://www.facebook.com/groups/413779162140585
Private Discussion Groups	
Vet Rehab	https://groups.io/g/VetRehab/topics
VIN Neurology Group	https://www.vin.com/members/boards/discussionviewer.aspx?documentid=12482199&SAId=5&MyActivities=1&f5=1&FindSince=26280000
Newsletters	
European College of Sports Medicine and Rehabilitation	
American College of Sports Medicine and Rehabilitation	
American Association of Rehabilitation Veterinarians	
Canine Arthritis Resources and Education	

### Statistical analysis

Descriptive and formal statistical analyses were conducted on the survey response data for canine and equine respondents separately. Univariate summary statistics of the key survey variables of interest were performed, which included frequency for categorical variables (e.g., place of employment, background education, training in axial examination) and statistical summaries for continuous variables (i.e., perceived prevalence) reported across the six clinical scenarios.

Additional descriptive analyses were conducted by either background education, whether respondents worked in academia, or examination techniques employed. The corresponding outcomes examined for these subgroup analyses included the perception of the prevalence of BPAD within the six clinical scenarios and the performance of specific examination techniques. ANOVA and *t*-tests (with corresponding 95% confidence intervals) were performed to answer the primary questions of interest, which included whether veterinarians from different education backgrounds varied in their perception of the prevalence of BPAD (ANOVA), whether those that had additional training were more likely to perceive the presence of BPAD (one-sided two-sample *t*-tests), and whether there was an increased perception of the prevalence of BPAD among those that examined individual vertebral motion segments or palpated for the presence of myofascial trigger points (two-sample *t*-tests). Majority opinion was determined from first quartile values to reflect the consensus of approximately 75% of respondents.

Scenario 1 described any patient with a musculoskeletal diagnosis. Scenarios 2 through 6 represented potential clinical manifestations of scenario 1 and were therefore considered subsets of scenario 1. To assess the effect of additional training, responses to scenario 1 and the average response to the 2–6 clinical scenarios were analyzed using one-sided two-sample *t*-tests.

The statistical significance level used for all statistical tests was *p* < 0.05. All statistical analyses were performed using the R programming language (Version 4.0.3) (76).

## Results

A total of 1,044 individuals responded to the survey, but only 1,000 met the inclusion criterion. Forty-four surveys were excluded due to the respondents not possessing a veterinary degree or for failing to provide replies for sections 2 and 3.

### Section 1: education background

Most respondents were canine practitioners (75%); 17% were equine, and 8% were mixed. Divided by employment description, 89% worked in non-academic settings and 11% worked in academia. By education background, 44% of respondents had a veterinary degree only (DVM). Boarded specialists included 15% veterinary surgeons (VS), 10% sports medicine and rehabilitation (VSMR), and 5% other (VSP). The majority (81%) of other boarded specialists (VSP) were canine neurologists. Respondents with certification included 12% in spinal manipulation therapy (CSMT), 11% in acupuncture (CVA), and 4% in rehabilitation (CCR).

Most canine respondents (80%) reported being taught how to perform a detailed appendicular examination in veterinary school, but only 54% reported being taught how to perform a detailed axial examination. Similarly, 87% of equine respondents reported being taught how to perform a detailed appendicular examination in veterinary school, but only 30% reported being taught how to perform a detailed axial examination.

Six percent of canine and 4% of equine practitioners that reported receiving training in axial examination during their traditional veterinary education, additionally specified that the training they received was inadequate. Thus, only 48% of canine and 26% of equine respondents reported being adequately taught how to perform an axial examination. Additionally, 2% of canine and 5% of equine respondents specifically expressed satisfaction with their veterinary education in axial examination techniques.

### Section 2: clinical scenarios

Overall responses for each of the 6 clinical scenarios (Table 2) are reported for both dogs (Table 4) and horses (Table 5). There was only one equine respondent in the other board specialty (VSP) category, so that category was eliminated from the equine results, and the respondent was included in the DVM group instead. These overall responses were then divided into categories based on respondents’ education background (Table 6; Supplementary Figure 1 for dogs; Table 7; Supplementary Figure 2 for horses), whether respondents did or did not work in academia (Table 8 for dogs; Table 9 for horses), and examination techniques (Tables 10, 11).

**Table 4:** Percentage distribution of survey responses for scenarios 1 through 5 from canine practitioners identifying that the primary lesion is axial, appendicular, or both axial and appendicular such that the primary lesion cannot be determined by examination alone.

Lesion location	Minimum	1st quartile	Median	3rd quartile	Maximum	Mean	SD	Number of canine respondents
Scenario 1: prior musculoskeletal diagnosis								
Axial	0	20	25	40	100	30.1	18.5	450
Appendicular	2	50	60	79	99	60.1	21.1	453
Both	0	5	10	20	100	16.1	15.3	373
Scenario 2: unilateral limb lameness								
Axial	0	8	10	20	95	17.6	16.1	443
Appendicular	3	70	80	90	100	76.5	18.2	455
Both	0	5	10	10	100	11.2	12.5	318
Scenario 3: altered spinal posture								
Axial	0	69	80	90	100	73.6	23.5	448
Appendicular	0	5	10	25	95	19.1	20.4	431
Both	0	5	10	20	100	14.2	15.2	303
Scenario 4: reduced athletic performance								
Axial	3	30	50	70	99	49.2	24.3	381
Appendicular	0	20	40	50	95	38.9	24.4	374
Both	0	10	20	30	100	21.2	19.1	267
Scenario 5: no reported clinical signs								
Axial	0	10	20	40	95	26.1	23.0	387
Appendicular	0	10	20	30	100	22.3	19.8	384
Both	0	5	10	20	100	13.4	15.7	208
Scenario 6: concurrent with limb lameness								
Axial	0	15	30	75	100	42.6	32.0	443

Scenario 6 reports the percentage likelihood that a primary appendicular lesion also has BPAD.

**Table 5:** Distribution of survey responses of equine practitioners identifying the probability that, for scenarios 1 through 5, the primary lesion is axial, appendicular, or both axial and appendicular such that the primary lesion cannot be determined by examination alone.

Lesion location	Minimum	1st quartile	Median	3rd quartile	Maximum	Mean	SD	Number of equine respondents
Scenario 1: prior musculoskeletal diagnosis								
Axial	1	10	20	27.5	98	21.1	15.7	175
Appendicular	2	50	70	80	100	65.3	21.5	177
Both	0	5	10	20	75	18.1	15.9	152
Scenario 2: unilateral limb lameness								
Axial	0	5	10	20	95	13.4	13.9	165
Appendicular	5	70	80	90	100	79.8	17.7	173
Both	0	5	10	15	70	11.9	12.1	124
Scenario 3: altered spinal posture								
Axial	5	40	60	80	100	59.5	24.7	169
Appendicular	0	10	25	50	90	29.6	22.0	164
Both	0	10	15	20	100	20.7	20.0	130
Scenario 4: reduced athletic performance								
Axial	1	25	40	60	100	43.8	23.8	159
Appendicular	0	20	33	50	95	38.4	22.2	156
Both	0	10	20	30	100	23.6	18.9	125
Scenario 5: no reported clinical signs								
Axial	0	20	30	50	100	38.0	26.4	149
Appendicular	0	10	20	50	100	29.1	21.5	148
Both	0	10	15	22.5	95	19.4	17.0	99
Scenario 6: concurrent with limb lameness								
Axial	0	32.5	60	80	100	58.4	29.4	171

Scenario 6 reports the percentage likelihood that a primary appendicular lesion also has an axial lesion.

**Table 6:** Responses from canine practitioners, divided by education background, describing the percentage of primary axial lesions for scenarios 1 through 5.

Education background	Minimum	Median	Maximum	Mean	SD	Number of canine respondents
Scenario 1: prior musculoskeletal diagnosis						
DVM	3	25	100	31.3	18.6	199
VS	1	15	95	20.7	17.5	66
VSMR	5	30	65	30.0	13.3	32
VSP	0	20	95	39.6	31.9	12
CCR	5	20	50	25.1	12.4	22
CVA	1	29.5	90	30.9	17.3	62
CSMT	4	33	85	36.1	17.8	57
COMBINED	0	25	100	30.1	18.5	450
Scenario 2: unilateral limb lameness						
DVM	0	10	95	17.5	16.3	193
VS	0	10	95	12.0	13	68
VSMR	2	10	75	17.0	15	31
VSP	10	22.5	95	38.8	28.9	12
CCR	2	10	50	17.3	14.8	23
CVA	0	20	60	18.4	12.5	61
CSMT	2	18	95	19.9	15.8	55
COMBINED	0	10	95	17.6	16.1	443
Scenario 3: altered spinal posture						
DVM	0	80	100	76.8	22.9	194
VS	5	85	100	76.0	26.1	65
VSMR	8	50	95	52.2	24.6	31
VSP	30	90	99	86.3	19.3	11
CCR	20	80	100	76.6	19.3	24
CVA	10	80	100	69.9	22.3	64
CSMT	20	75	100	72.4	18.3	59
COMBINED	0	80	100	73.6	23.5	448
Scenario 4: reduced athletic performance						
DVM	5	50	95	46.7	23	153
VS	5	40	90	42.6	24.2	63
VSMR	15	50	90	50.4	23.6	27
VSP	10	50	95	45.0	25.1	9
CCR	5	32.5	90	38.9	24.3	22
CVA	5	50	99	51.9	25.1	52
CSMT	3	70	98	65.2	21	55
COMBINED	3	50	99	49.2	24.3	381
Scenario 5: no reported clinical signs						
DVM	0	10	80	18.4	18.3	170
VS	0	10	10	22.3	18.4	11
VSMR	5	35	22.5	27.5	17.3	20
VSP	1	10	30	34.4	23.7	56
CCR	0	22.5	10	18.3	20.2	48
CVA	1	30	40	42.2	26.4	57
CSMT	2	40	35	38.7	24.6	25
COMBINED	0	50	20	26.1	23	387
Scenario 6: concurrent with limb lameness						
DVM	1	22.5	100	29.8	26.8	192
VS	0	12.5	100	19.7	20.5	66
VSMR	10	80	100	69.0	23.8	31
VSP	10	30	90	38.7	29.2	11
CCR	1	30	100	42.4	30.5	23
CVA	5	77.5	100	66.2	30.2	62
CSMT	10	80	100	72.8	24.9	58
COMBINED	0	30	100	42.6	32	443

Scenario 6 reports the percentage likelihood that a primary appendicular lesion also has an axial lesion. DVM, veterinarian with no additional specializations or certifications; VS, surgical specialist; VSMR, sports medicine and rehabilitation specialist; VSP, veterinary specialist in field outside of surgery or sports medicine; CCR, certified in canine rehabilitation; CVA, certified in veterinary acupuncture; CSMT, certified in spinal manipulation.

**Table 7:** Responses from equine practitioners, divided by education background, describing the percentage of primary axial lesions for scenarios 1 through 5.

Education background	Minimum	Median	Maximum	Mean	SD	Number of canine respondents
Scenario 1: prior musculoskeletal diagnosis						
DVM	2	15	30	15.4	8.7	47
VS	4	15	50	17.6	11.5	30
VSMR	5	23	85	25.4	15.9	43
VSP	NA	NA	NA	NA	NA	NA
CCR	NA	NA	NA	NA	NA	NA
CVA	1	15	30	14.3	8.3	15
CSMT	5	25	98	28.3	21.7	40
COMBINED	1	20	98	21.1	15.7	175
Scenario 2: unilateral limb lameness						
DVM	0	5	30	8.8	6.8	44
VS	1	10	30	10.0	6.4	30
VSMR	2	15	33	15.1	8.6	42
VSP	NA	NA	NA	NA	NA	NA
CCR	NA	NA	NA	NA	NA	NA
CVA	0	10	90	16.5	23.3	13
CSMT	0	10	95	18.5	21.7	36
COMBINED	0	10	95	13.4	13.9	165
Scenario 3: altered spinal posture						
DVM	5	60	100	51.3	24.7	45
VS	5	50	100	52.0	24.9	30
VSMR	20	70	100	66.0	23.2	42
VSP	NA	NA	NA	NA	NA	NA
CCR	NA	NA	NA	NA	NA	NA
CVA	30	70	90	63.6	21.0	11
CSMT	25	70	100	66.4	23.6	41
COMBINED	5	60	100	59.5	24.7	169
Scenario 4: reduced athletic performance						
DVM	5	30	90	36.0	22.0	42
VS	2	30	90	37.9	23.2	26
VSMR	15	45	95	47.6	22.0	39
VSP	NA	NA	NA	NA	NA	NA
CCR	NA	NA	NA	NA	NA	NA
CVA	1	40	70	40.7	19.6	13
CSMT	10	50	100	53.3	25.9	39
COMBINED	1	40	100	43.8	23.8	159
Scenario 5: no reported clinical signs						
DVM	0	20	80	25.3	19.0	39
VS	0	35	85	33.6	25.5	21
VSMR	10	40	100	40.3	24.8	37
VSP	NA	NA	NA	NA	NA	NA
CCR	NA	NA	NA	NA	NA	NA
CVA	10	20	80	30.4	22.6	12
CSMT	10	50	100	52.8	28.8	40
COMBINED	0	30	100	38.0	26.4	149
Scenario 6: concurrent with limb lameness						
DVM	0	50	100	45.2	26.8	42
VS	1	30	80	36.9	28.2	30
VSMR	15	50	95	57.5	23.2	40
VSP	NA	NA	NA	NA	NA	NA
CCR	NA	NA	NA	NA	NA	NA
CVA	30	90	100	82.3	18.3	15
CSMT	10	85	100	78.4	22.4	44
COMBINED	0	60	100	58.4	29.4	171

Scenario 6 reports the percentage likelihood that a primary appendicular lesion also has an axial lesion. DVM, veterinarian with no additional specializations or certifications; VS, surgical specialist; VSMR, sports medicine and rehabilitation specialist; VSP, veterinary specialist in field outside of surgery or sports medicine; CCR, certified in canine rehabilitation; CVA, certified in veterinary acupuncture; CSMT, certified in spinal manipulation.

**Table 8:** Responses from canine practitioners, divided by whether or not they work in academia, describing the percentage of primary axial lesions for scenarios 1 through 5.

Employment	Minimum	Median	Maximum	Mean	SD	Number of canine respondents
Scenario 1: prior musculoskeletal diagnosis						
Academia	0	20	75	24.8	16.6	40
Non-academia	1	25	100	30.6	18.6	410
Scenario 2: unilateral limb lameness						
Academia	2	10	60	13.5	11.8	40
Non-academia	0	15	95	18.0	16.4	403
Scenario 3: altered spinal posture						
Academia	5	80	100	66.6	30.1	39
Non-academia	0	80	100	74.3	22.7	409
Scenario 4: reduced athletic performance						
Academia	5	40	90	41.1	22.9	36
Non-academia	3	50	99	50.0	24.4	345
Scenario 5: no reported clinical signs						
Academia	0	20	80	27.5	24.6	28
Non-academia	0	20	95	26.0	22.9	359
Scenario 6: concurrent with limb lameness						
Academia	1	20	100	32.9	31.7	40
Non-academia	0	30	100	43.6	31.9	403

Scenario 6 reports the percentage likelihood that a primary appendicular lesion also has an axial lesion.

**Table 9:** Responses from equine practitioners, divided by whether or not they work in academia, describing the percentage of primary axial lesions for scenarios 1 through 5.

Employment	Minimum	Median	Maximum	Mean	SD	Number of canine respondents
Scenario 1: prior musculoskeletal diagnosis						
Academia	4	20	85	21.9	15.4	33
Non-academia	1	20	98	20.9	15.8	142
Scenario 2: unilateral limb lameness						
Academia	2	10	30	13.0	7.1	32
Non-academia	0	10	95	13.5	15.1	133
Scenario 3: altered spinal posture						
Academia	10	50	100	55.7	25.4	31
Non-academia	5	60	100	60.4	24.5	138
Scenario 4: reduced athletic performance						
Academia	5	50	95	47. 8	25.2	27
Non-academia	1	40	100	43.0	23.5	132
Scenario 5: no reported clinical signs						
Academia	10	45	100	46	24.0	20
Non-academia	0	30	100	36.7	26.6	129
Scenario 6: concurrent with limb lameness						
Academia	1	50	90	49.1	26.5	32
Non-academia	0	70	100	60.6	29.7	139

Scenario 6 reports the percentage likelihood that a primary appendicular lesion also has an axial lesion.

**Table 10:** Comparison of axial skeletal examination techniques for canine and equine practitioners, divided by whether they work in academia, and divided by education background.

Canine and equine examination of each individual vertebra																								
	Canine examination of each individual vertebra												Equine examination of each individual vertebra											
	Overall		Resting Position		PROM		AROM		End Feel		Pain		Overall		Resting Position		PROM		AROM		End Feel		Pain	
	*N*	%	*N*	%	*N*	%	*N*	%	*N*	%	*N*	%	*N*	%	*N*	%	*N*	%	*N*	%	*N*	%	*N*	%
Overall	246	52.3%	195	41.5%	160	34.0%	131	27.9%	117	24.9%	232	49.4%	104	55.6%	92	49.2%	93	49.7%	78	41.7%	70	37.4%	101	54.0%
Academia	23	52.3%	19	43.2%	17	38.6%	10	22.7%	12	27.3%	23	52.3%	17	51.5%	12	36.4%	14	42.4%	15	45.5%	12	36.4%	17	51.5%
Non-academia	223	52.3%	176	41.3%	143	33.6%	121	28.4%	105	24.6%	209	49.1%	87	56.5%	80	51.9%	79	51.3%	63	40.9%	58	37.7%	84	54.5%
DVM	75	36.8%	50	24.5%	35	17.2%	30	14.7%	23	11.3%	68	33.3%	13	25.0%	10	19.2%	7	13.5%	6	11.5%	5	9.6%	12	23.1%
VS	32	43.8%	27	37.0%	18	24.7%	12	16.4%	9	12.3%	31	42.5%	8	25.8%	7	22.6%	7	22.6%	6	19.4%	7	22.6%	8	25.8%
VSMR	28	84.9%	22	66.7%	24	72.7%	21	63.6%	22	66.7%	27	81.8%	33	75.0%	29	65.9%	31	70.5%	30	68.2%	18	40.9%	33	75.0%
VSP	5	38.5%	3	23.1%	3	23.1%	4	30.8%	1	7.7%	5	38.5%	NA	NA	NA	NA	NA	NA	NA	NA	NA	NA	NA	NA
CCR	8	34.8%	6	26.1%	6	26.1%	4	17.4%	4	17.4%	7	10.9%	NA	NA	NA	NA	NA	NA	NA	NA	NA	NA	NA	NA
CVA	40	62.5%	36	56.3%	24	37.5%	22	34.4%	11	17.2%	40	62.5%	7	50.0%	5	35.7%	5	35.7%	5	35.7%	2	14.3%	7	50.0%
CSMT	58	96.7%	51	85.0%	50	83.3%	38	63.3%	47	78.3%	54	90.0%	43	93.5%	41	89.1%	43	93.5%	31	67.4%	38	82.6%	41	89.1%

Canine and equine examination of the ribs																								
	Canine examination of the ribs												Equine examination of the ribs											
	Overall		Resting Position		PROM		AROM		End Feel		Pain		Overall		Resting Position		PROM		AROM		End Feel		Pain	
	*N*	%	*N*	%	*N*	%	*N*	%	*N*	%	*N*	%	*N*	%	*N*	%	*N*	%	*N*	%	*N*	%	*N*	%
Overall	187	39.9%	130	27.7%	64	13.7%	35	7.5%	59	12.6%	176	37.5%	98	52.4%	77	41.2%	49	26.2%	32	17.1%	36	19.3%	92	49.2%
Academia	13	29.5%	10	22.7%	7	15.9%	0	0.0%	7	15.9%	12	27.3%	11	33.3%	8	24.2%	4	12.1%	4	12.1%	3	9.1%	27	81.8%
Non-academia	174	40.8%	120	28.2%	57	13.4%	35	8.2%	52	12.2%	164	38.5%	87	56.5%	69	44.8%	45	29.2%	28	18.2%	33	21.4%	135	87.7%
DVM	49	24.1%	28	13.8%	6	3.0%	3	1.5%	15	7.4%	47	23.2%	16	30.8%	15	28.8%	4	7.7%	3	5.8%	2	3.9%	42	80.8%
VS	17	23.3%	11	15.1%	2	2.7%	2	2.7%	1	1.4%	12	16.4%	11	35.5%	4	12.9%	2	6.5%	3	9.7%	1	3.2%	24	77.4%
VSMR	25	75.8%	20	60.6%	15	45.5%	3	9.1%	13	39.4%	23	69.7%	22	50.0%	16	36.4%	9	20.4%	3	6.8%	8	18.2%	39	88.6%
VSP	6	46.2%	3	23.1%	1	7.7%	0	0.0%	0	0.0%	5	38.5%	NA	NA	NA	NA	NA	NA	NA	NA	NA	NA	NA	NA
CCR	10	43.5%	7	30.4%	1	4.3%	0	0.0%	0	0.0%	9	39.1%	NA	NA	NA	NA	NA	NA	NA	NA	NA	NA	NA	NA
CVA	30	46.9%	18	28.1%	12	18.8%	5	7.8%	4	6.3%	30	46.9%	5	35.7%	3	21.4%	1	7.1%	1	7.1%	2	14.3%	13	92.9%
CSMT	51	85.0%	43	71.7%	27	45.0%	22	36.7%	26	43.3%	50	83.3%	44	95.7%	39	84.8%	33	71.7%	22	47.8%	23	50.0%	44	95.7%

Canine and equine examination of the sacroiliac joints																								
	Canine examination of the sacroiliac joints												Equine examination of the sacroiliac joints											
	Overall		Resting Position		PROM		AROM		End Feel		Pain		Overall		Resting Position		PROM		AROM		End Feel		Pain	
	*N*	%	*N*	%	*N*	%	*N*	%	*N*	%	*N*	%	*N*	%	*N*	%	*N*	%	*N*	%	*N*	%	*N*	%
Overall	368	78.5%	260	55.4%	186	39.7%	114	24.3%	146	31.1%	350	74.6%	171	91.4%	147	78.6%	123	65.8%	97	51.9%	63	33.7%	162	86.6%
Academia	34	77.3%	24	54.5%	21	47.7%	9	20.4%	12	27.3%	34	77.3%	31	93.9%	24	72.7%	21	63.6%	22	66.7%	7	21.2%	27	81.8%
Non-academia	334	78.6%	236	55.5%	165	38.8%	105	24.7%	134	31.5%	316	74.4%	140	90.9%	123	79.9%	102	66.2%	75	48.7%	56	36.4%	135	87.7%
DVM	149	73.4%	92	45.3%	63	31.0%	45	22.2%	47	23.2%	141	69.5%	44	84.6%	36	69.2%	19	36.5%	11	21.1%	5	9.6%	42	80.8%
VS	56	76.7%	36	49.3%	20	27.4%	17	23.3%	13	17.8%	54	74.0%	26	83.9%	17	54.8%	15	48.4%	17	54.8%	4	12.9%	24	77.4%
VSMR	32	97.0%	23	69.7%	22	66.7%	10	30.3%	23	69.7%	31	93.9%	42	95.5%	39	88.6%	38	86.4%	33	75.0%	13	29.5%	39	88.6%
VSP	7	53.8%	6	46.2%	3	23.1%	1	7.7%	1	7.7%	7	53.8%	NA	NA	NA	NA	NA	NA	NA	NA	NA	NA	NA	NA
CCR	17	73.9%	9	39.1%	6	26.1%	2	8.7%	4	17.4%	16	69.6%	NA	NA	NA	NA	NA	NA	NA	NA	NA	NA	NA	NA
CVA	49	76.6%	39	60.9%	28	43.8%	10	15.6%	16	25.0%	45	70.3%	13	92.9%	12	85.7%	11	78.6%	6	42.9%	4	28.6%	13	92.9%
CSMT	60	100.0%	55	91.7%	44	73.3%	29	48.3%	42	70.0%	56	93.3%	46	100.0%	43	93.5%	40	87.0%	30	65.2%	37	80.4%	44	95.7%

**Table 11:** Comparison of myofascial examination techniques for canine and equine practitioners, divided by whether they work in academia, and divided by education background.

Canine examination of paraspinal musculature										
	Overall		Mass		Tone		Myalgia		MFTP	
	Number	Percentage	Number	Percentage	Number	Percentage	Number	Percentage	Number	Percentage
Overall	463	98.9%	437	93.4%	348	74.4%	404	86.3%	217	46.4%
Academia	44	95.7%	41	89.1%	33	71.7%	36	78.3%	18	39.1%
Non-academia	419	99.3%	396	93.8%	315	74.6%	368	87.2%	199	47.2%
DVM	199	98.5%	188	93.1%	133	65.8%	169	83.7%	47	23.3%
VS	72	98.6%	68	93.2%	44	60.3%	57	78.1%	15	20.5%
VSMR	33	100.0%	30	90.9%	31	93.9%	31	93.9%	29	87.9%
VSP	13	100.0%	13	100.0%	10	76.9%	12	92.3%	2	15.4%
CCR	23	100.0%	23	100.0%	20	87.0%	23	100.0%	19	82.6%
CVA	63	98.4%	58	90.6%	54	84.4%	56	87.5%	53	82.8%
CSMT	60	100.0%	57	95.0%	56	93.3%	56	93.3%	52	86.7%

Equine examination of paraspinal musculature										
	Overall		Mass		Tone		Myalgia		MFTP	
	Number	Percentage	Number	Percentage	Number	Percentage	Number	Percentage	Number	Percentage
Overall	187	100.0%	185	98.9%	165	88.2%	173	92.5%	102	54.5%
Academia	33	100.0%	33	100.0%	28	84.8%	30	90.9%	13	39.4%
Non-academia	154	100.0%	154	100.0%	137	89.0%	143	92.9%	89	57.8%
DVM	52	100.0%	52	100.0%	44	84.6%	47	90.4%	15	28.8%
VS	31	96.9%	30	93.8%	21	65.6%	26	81.3%	5	15.6%
VSMR	44	100.0%	44	100.0%	42	95.5%	44	100.0%	30	68.2%
CVA	14	93.3%	13	86.7%	14	93.3%	14	93.3%	12	80.0%
CSMT	46	100.0%	46	100.0%	44	95.7%	42	91.3%	40	87.0%

Across all 6 clinical scenarios, the range between the maximum and minimum values was at least 95%. For scenarios 1 through 5, the standard deviation ranged from 13 to 24% for canine respondents, and 12 to 25% for equine respondents. For scenario 6 (prevalence of primary limb lameness and secondary BPAD), the standard deviation increased to 32% (canine) and 29% (equine).

The majority of canine respondents report that a primary axial lesion can be found in at least 69% of patients presented with altered vertebral column posture and movement-related pain, 30% of patients presented with a non-specific reduction in athleticism, 20% of patients diagnosed with a musculoskeletal injury, 10% of patients with no reported clinical signs, and 8% of patients presented with unilateral limb lameness. Additionally, the majority agreed that BPAD can be found as a secondary or concurrent issue in at least 15% of cases presented with primary appendicular lameness.

The majority of equine respondents report that primary BPAD occurs in at least 40% of patients presented with altered vertebral column posture and saddle placement aversion, 25% of patients presented with a non-specific reduction in athletic performance, 20% of patients with no reported clinical signs, 10% of patients presented with an overall diagnosis of musculoskeletal injury, and in 5% of patients presented with unilateral limb lameness. Additionally, BPAD can be found in at least 33% of cases presented with primary appendicular lameness.

For scenarios 1 through 5, canine respondents reported that 11 to 21% of patients have concurrent axial and appendicular lesions that confound the clinicians’ ability to determine which is the primary and which is the secondary issue. Equine respondents reported a similar prevalence of 12 to 24% of concurrent axial and appendicular issues.

For scenario 1, on average, canine respondents reported a primary axial issue in 30% of patients presented with a musculoskeletal injury. Other veterinary specialists (VSP) reported the highest percentage (40%), and surgical specialists the lowest (20%). Equine respondents overall reported a lower prevalence of primary axial lesions (mean 20%), with spinal manipulation therapists and sports medicine specialists reporting a higher prevalence compared to other groups. The difference in opinion between education groups was statistically significant for both the canine (*p* < 0.001) and equine (*p* < 0.001) respondents.

In scenario 2, canine respondents reported a primary axial lesion in a mean of 18% of patients with a unilateral limb lameness. VSP reported a mean of 39%, whereas the remaining groups reported a mean ranging from 20% for spinal manipulation therapists to 12% for surgical specialists. Equine results were similar, with an overall mean of 13%. Acupuncturists reported the highest prevalence (18%), and surgical specialists the lowest (10%). The difference in opinion between education groups was statistically significant for both the canine (*p* < 0.001) and equine (*p* = 0.011) respondents.

For scenario 3a, canine respondents reported a primary axial lesion in a mean of 74% of patients presented with a species-specific clinical presentation that the authors felt was highly indicative of primary axial pain. Responses ranged from a mean of 86% for VSP, to 52% for sports medicine specialists. For scenario 3b, equine respondents averaged 59% for the presence of BPAD, with acupuncturists reporting the highest values (65%), and general practitioners reporting the lowest (51%). The difference in opinion between education groups was statistically significant for both the canine (*p* < 0.001) and equine (*p* = 0.006) respondents.

Scenario 4 consisted of patients presented with a non-specific reduction in athletic ability and no limb lameness. Canine respondents on average reported that 49% have a primary axial issue, and 39% have a primary appendicular issue. Responses ranged from a mean of 65% of spinal manipulation therapists to 39% of certified rehabilitation practitioners reporting a primary axial lesion. Equine respondents on average reported that 42% of such cases have a primary axial lesion, and 39% have a primary appendicular lesion. Responses ranged from a mean of 53% of spinal manipulation therapists reporting a primary axial lesion, to 36% of general practitioners reporting a primary axial lesion The difference in opinion between education groups was statistically significant for both the canine (*p* < 0.001) and equine (*p* = 0.007) respondents.

For scenario 5, on average, canine respondents reported a primary axial lesion in 26% of patients presented with no known signs of musculoskeletal pain, dysfunction, or reduced athletic performance. Spinal manipulation therapists and sports medicine specialists reported the highest (39 and 42% respectively) and general practitioners and surgical specialists reporting the lowest prevalence (18%). Equine respondents showed a similarly wide variation both within and between groups, with spinal manipulation therapists reporting the highest (53%) and general practitioners reporting the lowest prevalence (25%). The difference in opinion between groups was statistically significant for both the canine (*p* < 0.001) and equine (*p* < 0.001) respondents.

For scenario 6, on average, canine respondents reported secondary or concurrent axial lesions in 43% of patients presented with a primary limb lameness. Acupuncturists, spinal manipulation therapists, and sports medicine specialists reported means ranging from 66 to 72%, but general practitioners, VSP, and canine rehabilitation therapists reported means ranging from 30 to 42%. Surgical specialists reported a mean of 20%. Equine respondents demonstrated a similar dichotomy with acupuncturists and spinal manipulation therapists reporting 82 and 78% respectively, but lower values were reported by general practitioners, surgical specialists and sports medicine specialists (ranging from 37 to 58%). The difference in opinion between groups was statistically significant for both the canine (*p* < 0.001) and equine (*p* < 0.001) respondents.

When comparing canine responses between those with or without additional training, only spinal manipulation therapists appreciated a significantly greater prevalence of BPAD for patients presented with a musculoskeletal diagnosis (scenario 1). For equine respondents, both spinal manipulation therapists and sports medicine specialists reported an increased prevalence of BPAD for scenario 1. For combined scenarios 2 through 6, canine sports medicine specialists, acupuncturists, and spinal manipulation therapists reported significantly greater prevalence of BPAD, whereas canine rehabilitation therapists did not. Equine sports medicine specialists, acupuncturists, and spinal manipulation therapists also reported significantly greater BPAD for combined scenarios 2 through 6 relative to those without additional training (Table 12).

**Table 12:** One-sided two-sample *t*-tests comparisons between practitioners with and without additional training across scenario 1 and combined scenarios 2–6 for the perception of the prevalence of BPAD.

Education background	Scenario 1			Combined scenario 2–6		
	T value*	df	*p*-value	T value	df	*p*-value
Canine						
VSMR	−0.3	48.3	0.369	−3.4	38.0	0.001
CCR	1.4	30.1	0.915	−1.5	27.7	0.072
CVA	−0.7	99.6	0.237	−6.4	92.9	<0.001
CSMT	−2.6	86.3	0.005	−10.3	92.8	<0.001
Equine						
VSMR	−3.4	60.3	<0.001	−4.9	93.6	<0.001
CVA	0.8	22.5	0.785	−3.9	21.6	0.001
CSMT	−3.3	47.5	<0.001	−7.0	67.8	<0.001

VSMR, sports medicine and rehabilitation specialist; CCR, certified in canine rehabilitation; CVA, certified in veterinary acupuncture; CSMT, certified in spinal manipulation. Veterinarians without additional training includes those without additional certification or specialization (DVM); those with surgical specialization only (VS); and those with specialization in fields outside of VSMR or VS (VSP).

^*^Negative values indicate that the average prevalence reported by those with additional training in axial examination is greater than those without additional training (VS, VSP, DVM).

df, degrees of freedom.

### Section 3: axial examination

52% of canine respondents report examining individual vertebral motion segments, with the majority assessing for signs of pain (Table 10). Similarly, 56% of equine practitioners report examining individual vertebral motion segments, with the majority looking for signs of pain (Table 10). Among canine respondents, spinal manipulation therapists were most likely to examine individual vertebral motion segments (97%), followed by sports medicine specialists (85%). General practitioners and canine rehabilitation therapists were least likely to do so (37 and 35% respectively). A similar pattern emerged for equine respondents, with 93% of spinal manipulation therapists and 75% of sports medicine specialists examining individual vertebral motion segments, compared to only 26% of surgical specialists, and 25% of general practitioners.

Routine evaluation of the rib cage was performed by 40% of canine and 52% of equine respondents, primarily assessing for signs of pain (Table 10). For both canine and equine respondents, sports medicine specialists and spinal manipulation therapists were most likely to examine the ribs, whereas surgical specialists and general practitioners were least likely.

Examination of the sacroiliac joints occurred frequently; 91% of equine and 79% of canine respondents report routinely doing so. Pain was the most frequently assessed clinical sign. Among canine respondents, sports medicine specialists and spinal manipulation therapists were most likely to examine the sacroiliac joints (100 and 97% respectively). Greater than 93% of equine acupuncturists, spinal manipulation therapists and sports medicine specialists report examining the sacroiliac joints.

Examination of paraspinal musculature is routinely performed by 98% of canine respondents, most frequently assessing muscle mass (93%) and least frequently for myofascial trigger points (46%). One hundred percent of equine respondents reported examining the paraspinal musculature, with 99% assessing muscle mass and 55% palpating for myofascial trigger points. Under the category of “other,” 2 canine respondents reported assessing for heat, and 2 equine respondents reported palpating acupoints. Based on education categories, greater than 83% of canine sports medicine specialists, canine rehabilitation therapists, acupuncturists and spinal manipulation therapists palpate for myofascial trigger points, compared to fewer than 23% of general practitioners, surgical specialists, and other veterinary specialists. Equine respondents reported a similar dichotomy with greater than 80% of acupuncturists and spinal manipulation therapists palpating for myofascial trigger points, compared to fewer than 29% of general practitioners and surgical specialists.

Canine respondents that examined vertebral motion segments were significantly more likely to report primary axial lesions in patients with a known musculoskeletal diagnosis, and in those with no clinical history suggestive of musculoskeletal pathology (Table 13). They were also more likely to report detecting secondary axial issues in patients presented with a primary limb lameness. The difference in opinion between those practitioners that do and do not examine individual vertebral motion segments was not statistically significant for the remaining clinical scenarios. Equine respondents that examined vertebral motion segments were more likely to report primary axillary lesions for all 6 scenarios compared to those that do not (Table 13). This likelihood was statistically significant for all but scenario 4 (reduced athletic performance).

**Table 13:** Results of two-sample *t*-tests for each clinical scenario to assess whether practitioners that assess individual vertebral motion segments or palpate for the presence of myofascial trigger points are more likely to appreciate BPAD.

Examination technique	Canine respondents (*p*-values)	Equine respondents (*p*-values)
Scenario 1: prior musculoskeletal diagnosis		
Examine individual vertebral motion segments	0.009*	<0.001*
Palpate for myofascial trigger points	0.006*	0.033*
Scenario 2: unilateral limb lameness		
Examine individual vertebral motion segments	0.229	0.010*
Palpate for myofascial trigger points	0.035*	0.041*
Scenario 3: altered spinal posture		
Examine individual vertebral motion segments	0.611	<0.001*
Palpate for myofascial trigger points	<0.001*	0.004*
Scenario 4: reduced athletic performance		
Examine individual vertebral motion segments	0.080	0.070
Palpate for myofascial trigger points	0.008*	0.185
Scenario 5: no reported clinical signs		
Examine individual vertebral motion segments	0.080	<0.001*
Palpate for myofascial trigger points	<0.001*	0.004*
Scenario 6: concurrent with limb lameness		
Examine individual vertebral motion segments	<0.001*	<0.001*
Palpate for myofascial trigger points	<0.001*	<0.001*

*p*-value significance set at <0.05. Significant values are indicated by a “*” symbol.

Canine respondents that palpate for the presence of myofascial trigger points were significantly more likely to detect primary axial pathology for all 6 scenarios, compared to those that do not (Table 13). Equine respondents that palpate for the presence of myofascial trigger points were significantly more likely to detect primary axial pathology in 5 of 6 scenarios (Table 13).

## Discussion

### Education background

Our hypothesis that compared to canine veterinarians, equine veterinarians are more likely to receive education on how to perform an axial examination was not supported, nor was our hypothesis that equine veterinarians would report a higher prevalence of BPAD relative to canine veterinarians. We had speculated that the greater amount of equine BPAD research would increase the amount of education and awareness among equine practitioners relative to canine practitioners, but this theory was not supported by our findings.

Our hypothesis that the perception of BPAD prevalence would vary widely between veterinarians of different education backgrounds was supported. We did not hypothesize that there would be wide variation in opinion of veterinarians with the same education background, but the results suggest such disagreement exists. This disagreement is problematic, as it indicates a lack of consistency among veterinarians when diagnosing BPAD.

Our hypothesis that practitioners with additional training (sports medicine specialists, acupuncturists, and spinal manipulation therapists) would report a greater prevalence of BPAD compared to those without (general practitioners, surgical specialists, and other boarded specialists) was supported. However, our hypothesis that canine rehabilitation therapists would also report a greater prevalence of BPAD, was not supported; canine rehabilitation therapists agreed with general practitioners and non sports medicine specialists. Compared to those with additional training canine rehabilitation therapists were equally likely to palpate for myofascial trigger points, but less likely to examine individual vertebral motion segments (Table 10). Educators responsible for canine rehabilitation certification programs may wish to consider this information when developing future curricula.

### Clinical scenarios

Regardless of education background, the majority of respondents reported at least 20% of canine and 10% of equine patients with a prior diagnosis of musculoskeletal injury had a primary axial lesion, suggesting that primary BPAD is perceived to be a common phenomenon. The majority of respondents also report that primary axial lesions are found in at least 8% of canine and 5% of equine patients presented with unilateral appendicular lameness.

In horses, appendicular lameness has been attributed to primary cervical or costal pain, and to thoracolumbar or pelvic pain (10, 15–19). Outside of mechanical causes (e.g., intervertebral disc disease), canine lameness and sports medicine and rehabilitation textbooks make no reference to appendicular lameness resulting from an axial origin lesion (32, 52, 53). Further research is required to determine the significance and interrelationships between limb lameness and BPAD in both dogs and horses. Multiple researchers have suggested that axial issues such as myofascial trigger points, costal pain, or altered vertebral mobility are underdiagnosed causes of pain and lameness (30, 44–48).

The majority of equine respondents agreed that primary axial issues can be found in at least 25% of patients presented with a non-specific reduction in athletic ability (e.g., reduced jump height or speed) and no limb lameness. In horses, such decreases in athletic performance are recognized indicators of BPAD (13), but the authors are unaware of similar reports in dogs. Despite this, most canine respondents reported that least 30% of patients with reduced athletic performance have a primary axial lesion. This difference between the perceived likelihood that a primary axial lesion will result in non-specific reduced athletic performance in dogs, contrasted against the absence of published research on this topic, identifies another subject deserving of further investigation. Notably, both canine and equine respondents agreed that in such patients, primary axial lesions are more prevalent than primary appendicular lesions.

Most respondents reported that axial lesions occur in at least 10% of canine and 20% of equine patients with no presenting clinical complaint, which suggests that BPAD may escape detection by the owner or handler. Research suggests that up to 73% horse owners fail to identify lameness and 50% fail to report poor saddle fitting or back pain in their own horses (54). It is unknown if such patients are truly clinical sign free, or if they are instead expressing signs that the owners or handlers are not recognizing. It is also possible that the owners or handlers are recognizing clinical signs but attributing them to a different cause, such as a behavioral issue or a normal consequence of aging. Behavioral issues may be attributed to innate personality traits (“naughty” or “stubborn”) rather than being recognized as pain avoidance strategies (55).

Most respondents also reported that secondary or concurrent axial lesions can be identified in at least 15% of canine and 33% of equine patients with known appendicular lameness. These findings suggest that when treating limb lameness, examination of axial structures to rule out concurrent or secondary BPAD should be routinely performed.

### Veterinarian and patient caseload bias

There are many potential explanations for why such a wide variation in opinion exists between veterinarians, and because this research survey did not evaluate the reasons for this lack of agreement, the following comments are speculative. Biases are one possible explanation for the disagreement between veterinarians: biases of the individual veterinarian, and biases in the patient caseload presented to the veterinarian.

Differences in education on how to perform and interpret a detailed axial examination is likely one source of veterinarian bias. Most respondents report receiving inadequate formal veterinary education on how to perform an axial examination. This contrasts against the perception that axial lesions are common, and suggests that more should be done to educate veterinary students and practitioners. A widely held tenet is that a meticulous physical examination improves diagnostic sensitivity by facilitating detection of subtle abnormalities. By extension, practitioners not trained to perform detailed spinal or myofascial examinations may be at disadvantage for detecting BPAD compared to those with such training, leading to a perception that the prevalence of BPAD is lower.

Prior research has demonstrated that a veterinarian’s individual therapeutic skill set likely biases their perception of a given clinical presentation (56), meaning that when performing a physical examination, practitioners may specifically look for conditions that they possess the ability to treat. A surgical specialist’s physical examination may primarily focus on detecting pathology that requires surgical correction, instead of palpating for non-surgical conditions such as myofascial trigger points or non-painful vertebral hypomobility. Conversely, spinal manipulation therapists may perform a physical examination specifically directed toward detecting vertebral motion segment dysfunction, leading to different perceptions of prevalence of BPAD.

In addition to the bias created by differences in how a physical examination is performed, is the bias created by differences in ascribed importance of the examination findings. For example, surgical specialists may detect thoracolumbar hypomobility on examination, but dismiss this finding and instead focus attention on concurrent appendicular lesions requiring surgical intervention. Conversely, a sports medicine specialist treating patients at an international competition might ascribe greater significant to spinal stiffness or epaxial myalgia, due to the perceived effect of these conditions on athletic performance.

Veterinarian bias may affect other aspects of the diagnostic process beyond physical examination findings. An equine ambulatory practitioner that provides spinal manipulation therapy but does not perform diagnostic imaging, might report a higher prevalence of primary lumbar pain compared to an equine practitioner that routinely radiographs tarsal joints. Tarsal imaging can increase the likelihood of detecting tarsal pathology, and tarsal pathology has been linked to secondary back pain (57). Therefore, a practitioner detecting tarsal pathology more frequently may perceive greater prevalence of secondary BPAD, and a practitioner that detects tarsal pathology less frequently may perceive increased prevalence of primary BPAD.

Bias in caseload presentation is likely affected by practice focus, which in turn is affected by the veterinarian’s education background. For example, for some scenarios, those respondents with other speciality training (VSP) reported the greatest prevalence of primary BPAD, which may have been due to the high proportion of neurologists within that group. Neurologists are likely to work in an academic or specialist hospital setting where lameness cases are routinely seen initially by a surgical or sports medicine specialist, and only referred to a neurologist if an axial cause of lameness is suspected. Surgeons often reported a lower prevalence of BPAD, perhaps because patients referred to them have a suspected appendicular surgical lesion, and cases with suspected non-surgical BPAD are referred elsewhere - perhaps to a sports medicine specialist, increasing their perception of BPAD prevalence. Clients might also seek treatment from an acupuncture or spinal manipulation therapist as an adjunct treatment for a known appendicular lesion, thus increasing their perception of secondary BPAD.

Practice focus can also bias the makeup of owners or handlers, another group with a potentially varied perspective on the significance of physical examination findings. The handler of a high-level competition athlete may notice a reduction in the patient’s turning radius, a sign unlikely to be noticed by a recreational pet owner. Client demand may require that a clinician working with high level athletes identify and ascribe importance to subtle physical examination findings in a way that a pet owning client might not.

Linguistic, cultural, or geographic bias is another consideration. This survey was written in English, describing hypothetical scenarios. It was distributed through 24 different media groups, many of which were based in North America, and all were English speaking. A limitation of this survey was that it did not collect data on the respondents’ geographic location. Thus, it is unknown if national bias exists, and if so, how much such bias may have affected the respondents’ perspective. For example, there is likely substantial international and intercontinental variability between veterinary programs and clinical approaches. Such differences could affect respondents’ education background, individual bias, and caseload bias as described above. It is unknown whether the wording of the survey itself, particularly when describing non-standardized scenarios, added to the disagreement seen.

It was unknown if differences in perception would exist between veterinarians that do or do not work in an academic setting. As educators, veterinarians working in academia have greater opportunity to affect the perspectives of veterinary students and therefore of future veterinarians. Consequently, it was important to note whether the perceptions and examination techniques of academics and non-academics differed. Overall, differences in the perception of BPAD and in axial examination techniques employed varied little between those working or not working in academia, For each of the 6 clinical scenarios, non-academics consistently reported a slightly higher prevalence of BPAD and were more likely to examine the ribs and palpate for myofascial trigger points. However, there was no clear difference in the likelihood to examine individual vertebral motion segments or the sacroiliac joint, between those who do or do not work in academia.

### Definitions and terminology

Another contributing factor to the variation in veterinary perception of BPAD is the lack of standardized vocabulary to describe the wide array of clinical presentations, etiologies, and pathogeneses of BPAD. In human medicine, the term “non-specific back pain” has been used for 70 years as a broad descriptor of conditions that cause back pain yet lack specific findings on imaging or other testing (1, 8). Despite its widespread use for humans, the term ‘non-specific back pain’ is rarely used in veterinary medicine (31). However, human researchers argue that the term ‘non-specific back pain’ is problematic and misleading (1, 5, 8), and it does not include non-painful axial dysfunction (e.g., stiffness, muscle atrophy) within its definition. Therefore, we judge that the term ‘non-specific back pain’ is a poor choice for veterinarians to adopt. For this reason, we introduced the term “back pain and dysfunction (BPAD)” to fill this role. BPAD is not a diagnosis, but rather a syndrome that describes a spectrum of neuromusculoskeletal disorders that affect the axial region. Its presence may be determined through the combined collection of clinical or performance history, assessment of gait and mobility, physical examination findings, and diagnostic imaging results. The broad and potentially heterogenous scope of findings that may result from such data collection will likely necessitate the division of BPAD into clinically relevant subcategories based on chronicity, clinical severity, treatment indications, underlying mechanisms, physical examination findings, or pathoanatomic location, as is recommended for non-specific back pain (9, 58–60).

Currently, there is no standardized mechanism for canine practitioners to quantify, describe, or otherwise communicate the severity of BPAD in their patients, which may be a significant contributing factor to the wide discrepancy in perception. Lameness and myelopathy scales are routinely used to describe the presence and severity of limb lameness and spinal cord dysfunction (61, 62), but no BPAD grading scheme exists for veterinary patients. There are multiple pain scales described in horses, some of which include consideration of BPAD (13, 23, 63), but none of these scales appear to be widely used in routine clinical practice. The creation and widespread acceptance of a scale expressly designed to semi-quantify the severity of BPAD may lead to clearer communication between practitioners, which in turn might foster greater agreement on the prevalence of BPAD. The absence of such a scale will inherently hinder any future research on the prevalence or severity of BPAD.

### Axial examination

This survey investigated how respondents perform an axial examination, how this examination procedure varies with education background, and whether aspects of that examination affect the perception of BPAD prevalence. Our hypothesis that veterinarians who examine individual vertebral motion segments would report a higher prevalence of BPAD compared to veterinarians that do not, was supported. Canine respondents that examine individual vertebral motion segments were significantly more likely to appreciate primary axial lesions in 2 of 6 scenarios. Equine respondents that examined vertebral motion segments were significantly more likely to detect BPAD in 5 of 6 scenarios. It is unclear why this difference in examination techniques more consistently increased perception in horses than it did dogs.

These findings support the concept that a meticulous spinal examination increases the possibility of identifying clinical issues. In addition, the assessment of individual vertebral motion segments likely increases diagnostic precision. For example, a practitioner that palpates the spine as a whole may detect generalized pain, but someone who assesses each vertebral motion segment may detect focal pain and stiffness localized only to the T13-L1 vertebrae. For these reasons, we conclude that routine palpation of vertebral motion segments should be performed with every axial physical examination.

Routine evaluation of the rib cage was performed by 40% of canine and 52% of equine respondents, primarily to detect signs of pain. Dysfunction was assessed less frequently. The prevalence and clinical significance of palpable canine or equine rib pain and dysfunction is both unknown and controversial (15, 30). In humans, rib dysfunction is a documented caused of neck and shoulder pain (7), but remains largely overlooked in our veterinary patients. Canine research on this topic is confined to a case series of 3 dogs that demonstrated discomfort and lameness with no history of overt trauma, had palpable rib pain and dysfunction, and responded to appropriate therapy (30). In horses, forelimb lameness has been linked to cranial rib pathology evident on scintigraphy or radiography (15, 16). Caudal rib abnormalities identified on scintigraphy have been linked to resentment toward being saddled or ridden (13, 16).

The reason that so few respondents examined the rib cage is unknown, especially if one considers that quadrupeds ‘walk on their ribs’, meaning that the forelimbs are attached by a fibromuscular sling to the cranial rib cage, which then supports the weight and movement of the head, neck, trunk and associated thoracic and abdominal viscera (64). High impact forces are also transferred upward into the cranial rib cage when quadrupeds land from a jump on their thoracic limbs (65). Additionally, in horses, the saddle panel rest on the ribs, the girth or cinch is applied to the ribs, and leg aids are applied to the rib cage. The added weight of a saddle and rider places additional downward pressure over the back, which must be resisted by the rib cage at the costovertebral and costotransverse articulations (66). The potential existence of clinically significant pain resulting from canine or equine rib dysfunction is still speculative at this time; further research is required to determine what, if any, affect rib dysfunction has on patient comfort and mobility Because, examination of individual ribs is a low-risk diagnostic procedure that may increase the sensitivity of an axial physical examination, we recommend that it be routinely performed, even though the clinical significance of any abnormal physical examination findings must be interpreted cautiously until further research is performed.

Examination of the sacroiliac joint occurred frequently; 79% of canine and 91% of equine respondents report routinely doing so. Pain was the clinical sign most frequently assessed, followed by an altered neutral resting position, and passive joint range of motion. Sacroiliac joint pain associated with osteoarthritic changes has long been recognized in both humans and horses (3, 14). Radiographically-evident sacroiliac joint pathology is common in dogs (26, 67, 68). However, canine sacroiliac joint pain has not been identified (29). In horses, a definitive diagnosis of sacroiliac joint pain requires ruling out hind limb issues and other adjacent pathology (e.g., lumbosacral, intertransverse joints) and the use of diagnostic local anesthesia (69). Therefore, the term ‘lumbosacral or sacroiliac regional pain and dysfunction’ is often used in horses.

In dogs, performing diagnostic sacroiliac joint local anesthesia is technically challenging and not routinely performed in clinical practice (70). However, similar to horses, researchers report detecting sacroiliac joint region pain on palpation (71, 72). Three quarters of canine respondents report routinely palpating for sacroiliac joint pain when performing a musculoskeletal examination. Presumably, these veterinarians are being intermittently rewarded with a positive finding, or they would not invest time looking for sacroiliac joint pain and this behavior would undergo extinction.

Further research is needed to determine the prevalence of sacroiliac joint pain in dogs, and to validate the techniques that best differentiate sacroiliac joint pain from pain of adjacent structures. Given that evidence suggests that sacroiliac joint pathology is common in veterinary patients, that it is likely to contribute to repetitive use and secondary pathology of neighboring structures, and that palpation of the sacroiliac joint can elicit a pain response in affected animals (4, 14, 26, 67, 71, 72), we conclude that sacroiliac joint palpation should be routinely performed with every axial skeletal examination.

Our hypothesis that those clinicians who palpate for the presence of myofascial trigger points appreciate a greater prevalence of BPAD relative to those who do not, was supported. Respondents who palpate for myofascial trigger points reported a significantly greater prevalence of primary BPAD across all 6 scenarios (canine), and 5 out of 6 scenarios (equine).

Multiple studies have reported that myofascial trigger points are both a common and underdiagnosed cause of pain (44–49). Therefore, we suggest that myofascial trigger point palpation should be routinely performed with every musculoskeletal examination.

Although vertebral motion segment palpation was performed by approximately half the respondents in this survey, its inherently subjective nature, coupled with its use on patients incapable of verbally expressing their nociceptive experience, confounds our ability to perform quantitative or repeatable research to determine the validity of examination techniques. This is similarly true of many appendicular examination techniques that are routinely taught to, and employed by, veterinarians globally. Validation research on axial examination techniques should be performed, but its current absence does not preclude their use, particularly since they are low risk and rely on principles routinely applied both to veterinary appendicular orthopedic examination, and to examination of the human axial skeleton.

The findings of this survey did not prove that assessment of individual vertebral motion segments or for myofascial trigger points increases the sensitivity of an axial examination, but it indirectly supports that conclusion. This, coupled with an absence of evidence suggesting that examining individual vertebral motion segments, ribs, sacroiliac joints, and paraspinal musculature will cause the patient harm, suggests that doing so should be included with every axial skeletal examination. Moving forward, researchers investigating topics related to BPAD should specify in their methodology the physical examination techniques that were employed when making axial assessments.

### Limitations

A limitation of this study is that the surveyed population is unlikely to reflect the broader veterinary population; over half of survey respondents were either veterinary specialists, or possessed certification in rehabilitation, acupuncture, or spinal manipulation therapy, and thus are likely overrepresented compared to the general veterinary population. This disproportionality is likely a reflection of the social media outreach described in Table 3; only 9 of the 24 groups that advertised this research catered to a general practitioner audience, but 7 of the groups catered to veterinarians with an interest in rehabilitation, sports medicine, or manual therapy. It is likely that the distribution of this survey within those groups favored participation by clinicians with a particular interest in BPAD. Such increased interest might affect clinical philosophies and interpretive approaches in a way that this survey did not measure. The sample of survey respondents self-selected whether to participate, which also creates respondent bias.

Because of the way this survey was advertised, the response rate impossible to calculate. Although this survey distribution format was successful from the perspective that it generated a large number of responses, the authors would have preferred to see a greater proportion of general practitioners participating.

To avoid influencing responses, the authors elected not to define specific terms. It was anticipated that respondents would differ in how they distinguished clinically significant BPAD, or in the criteria to distinguish between primary and secondary lesions. Imposing rigid definitions describing how to make such distinctions would have potentially introduced bias, in part because universally accepted terminology or diagnostic criteria to categorize the existence or severity of BPAD does not exist in veterinary patients.

Future veterinary research and interprofessional communication would benefit from the development of standardized terminology that categorizes BPAD based on severity. Additionally, achieving broad agreement on how a physical examination of the axial region should be performed, would also benefit both clinicians and researchers.

The majority of respondents indicated that for scenarios 1 through 5, 5 to 10% of patients present with concurrent axial and appendicular musculoskeletal lesions, such that respondents were unable to determine which lesion was the primary cause of the reported clinical signs, or if both lesions contributed concurrently. These comorbid presentations were not used to calculate the prevalence of primary axial lesions. This exclusion increases clarity, but likely results in mild under-reporting in the actual perception of significant BPAD.

Additionally, many respondents either failed to answer all questions, or responded by stating that they did not know the answer. The reasons for this shortfall were not explored. Blank or “do not know” responses were excluded from data analysis. An added comment section for respondents to explain their responses or lack thereof would have strengthened the study. Future research could examine whether the prevalence of BPAD differs between equine and canine populations, or whether the variation in perception of BPAD prevalence would improve with the addition of standardized examination and reporting methods.

The perspectives gained from these clinical scenarios, coupled with information on how physical examinations are performed has produced preliminary data from which future studies can be designed. The reported prevalence of BPAD in this study should not be conflated with the actual prevalence, which remains unknown. Veterinarians of different education backgrounds showed significant disagreement, but no conclusions about the superiority of one group’s findings over another’s can be inferred.

This study provides the first large-scale assessment of veterinary perceptions regarding the prevalence of back pain and dysfunction (BPAD) in dogs and horses and highlights substantial variability in clinical opinion across practitioners, specialties, and examination approaches. Although the actual prevalence of BPAD remains unknown, respondents consistently perceived axial disorders to be common contributors to musculoskeletal pain, reduced performance, and even unilateral limb lameness. Veterinarians with additional training in sports medicine, acupuncture, or spinal manipulation, as well as those who routinely assessed vertebral motion segments and myofascial trigger points, reported a significantly greater prevalence of BPAD, suggesting that education and examination methodology strongly influence clinical recognition of axial disorders. These findings align with growing evidence from both veterinary and human medicine that axial dysfunction is multifactorial, frequently overlooked, and often difficult to localize using conventional diagnostic approaches. Importantly, the results emphasize the need for standardized terminology, validated physical examination protocols, and objective outcome measures to improve consistency among clinicians. Future work should focus on establishing reliable methods for identifying and quantifying BPAD, developing standardized reporting systems, and conducting prospective studies that correlate physical examination findings with diagnostic imaging, functional performance measures, treatment outcomes, and longitudinal follow-up. Such efforts will be essential to move the field from subjective perception toward evidence-based determination of the true prevalence, clinical significance, and optimal management of BPAD in veterinary patients.

## Conclusion

The actual prevalence of BPAD remains unknown, and perceptions on its prevalence vary widely. Respondents with sports medicine specialty status, or certification in either spinal manipulation or acupuncture therapy, are more likely to perceive the existence of BPAD compared to specialists in other disciplines, general practitioners, or those certified in canine rehabilitation therapy.

Most veterinarians agree that primary axial lesions are common among patients diagnosed with a musculoskeletal condition, and in patients presented with unilateral limb lameness. Primary axial lesions were deemed more prevalent than primary appendicular lesions in patients presented for a non-specific reduction in athletic performance. BPAD was also frequently reported in patients with no history of musculoskeletal pain, and as a secondary or coincidental finding in patients with a primary limb lameness.

Respondents who examine individual vertebral motion segments or that palpate for myofascial trigger points are more likely to detect BPAD. Although controversy exists regarding the reliability and diagnostic reproducibility of these examination techniques, and there is broad agreement that more research is needed, such techniques are low risk and minimally invasive to the patient. Therefore, despite the aforementioned controversies, we conclude that an axial skeletal examination should include palpation of individual vertebral motion segments, the ribs, and the sacroiliac joints - assessing for pain, range of motion, end feel, and neutral resting position. The myofascial component of the axial examination should include palpation for myofascial trigger points, as well as assessment of muscle mass, tone, and myalgia.

This research highlights the need for a standardized format when performing an axial physical examination, and when reporting clinical findings. Developing a standardized reporting system, such as a back pain and dysfunction scale, could potentially improve communication between practitioners and improve the quality of future research.

## Data Availability

The raw data supporting the conclusions of this article will be made available by the authors, without undue reservation.

## Footnotes

1
www.surveymonkey.com

## Supplementary material

SUPPLEMENTARY FIGURE 1 Box plot of responses from canine practitioners for each clinical presentation divided by education background, depicting the percentage of primary axial lesions for scenarios 1 through 5. Scenario 6 reports the percentage likelihood that a primary appendicular lesion also has an axial lesion. DVM, veterinarian with no additional specializations or certifications; VS, surgical specialist; VSMR, sports medicine and rehabilitation specialist; VSP, veterinary specialist in field outside of surgery or sports medicine; CCR, certified in canine rehabilitation; CVA, certified in veterinary acupuncture; CSMT, certified in spinal manipulation.

SUPPLEMENTARY FIGURE 2 Box plot of responses from equine practitioners for each clinical presentation divided by education background, depicting the percentage of primary axial lesions for scenarios 1 through 5. Scenario 6 reports the percentage likelihood that a primary appendicular lesion also has an axial lesion. DVM, veterinarian with no additional specializations or certifications; VS, surgical specialist; VSMR, sports medicine and rehabilitation specialist; VSP, veterinary specialist in field outside of surgery or sports medicine; CCR, certified in canine rehabilitation; CVA, certified in veterinary acupuncture; CSMT, certified in spinal manipulation.
