# Supplementary material for: Factors affecting veterinary perception of the prevalence of back pain and dysfunction in dogs and horses
Source: Front Vet Sci. 2026 Aug 17;13:1857696. doi: 10.3389/fvets.2026.1857696 (PMC13528434; doi:10.3389/fvets.2026.1857696)
Supplement: Supplementary file 2 [file Data_Sheet_2.PDF]

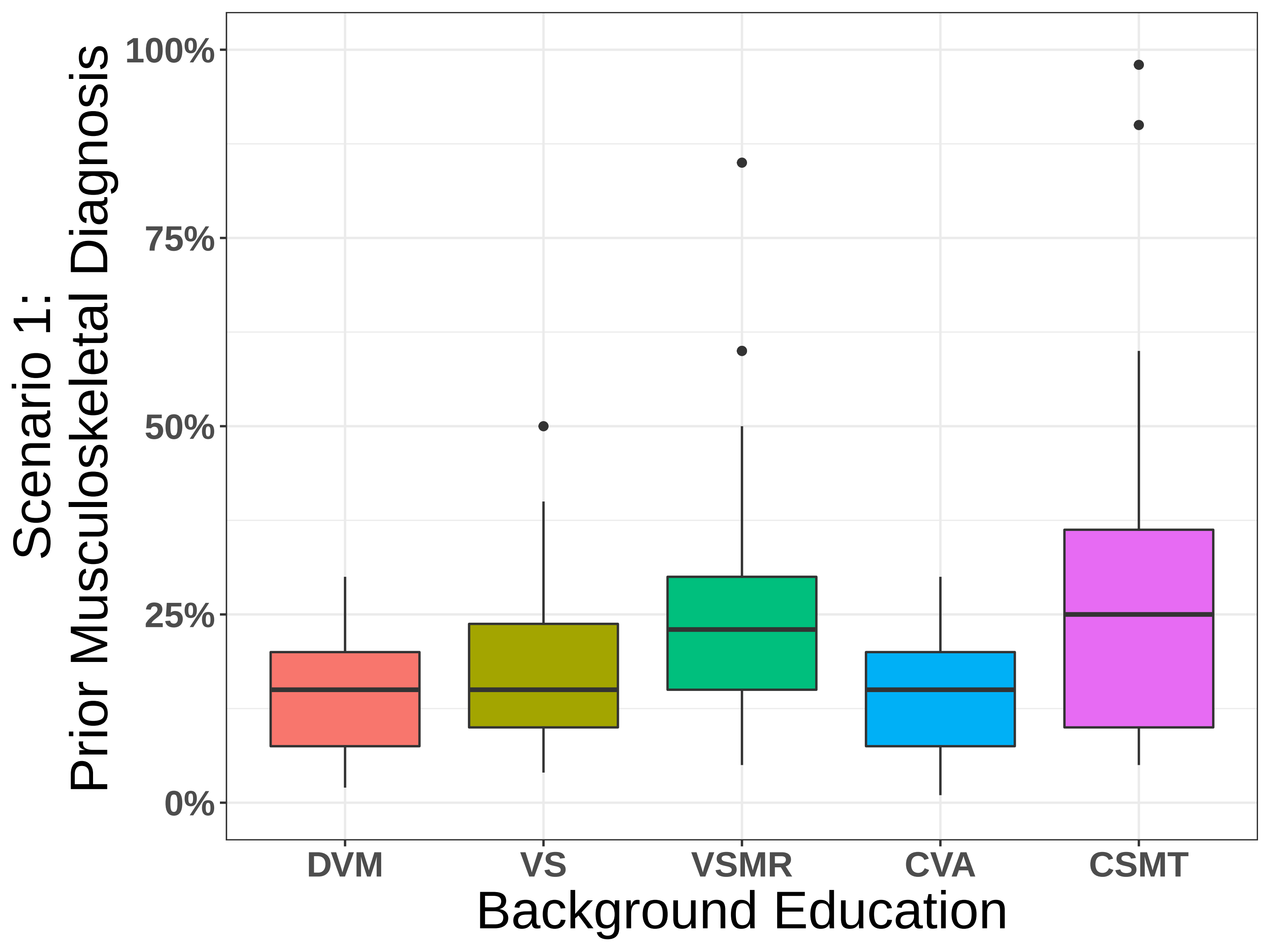

Scenario 1:
Prior Musculoskeletal Diagnosis
100%
75%
50%
25%
0%
DVM
VS
VSMR
CVA
CSMT
Background Education

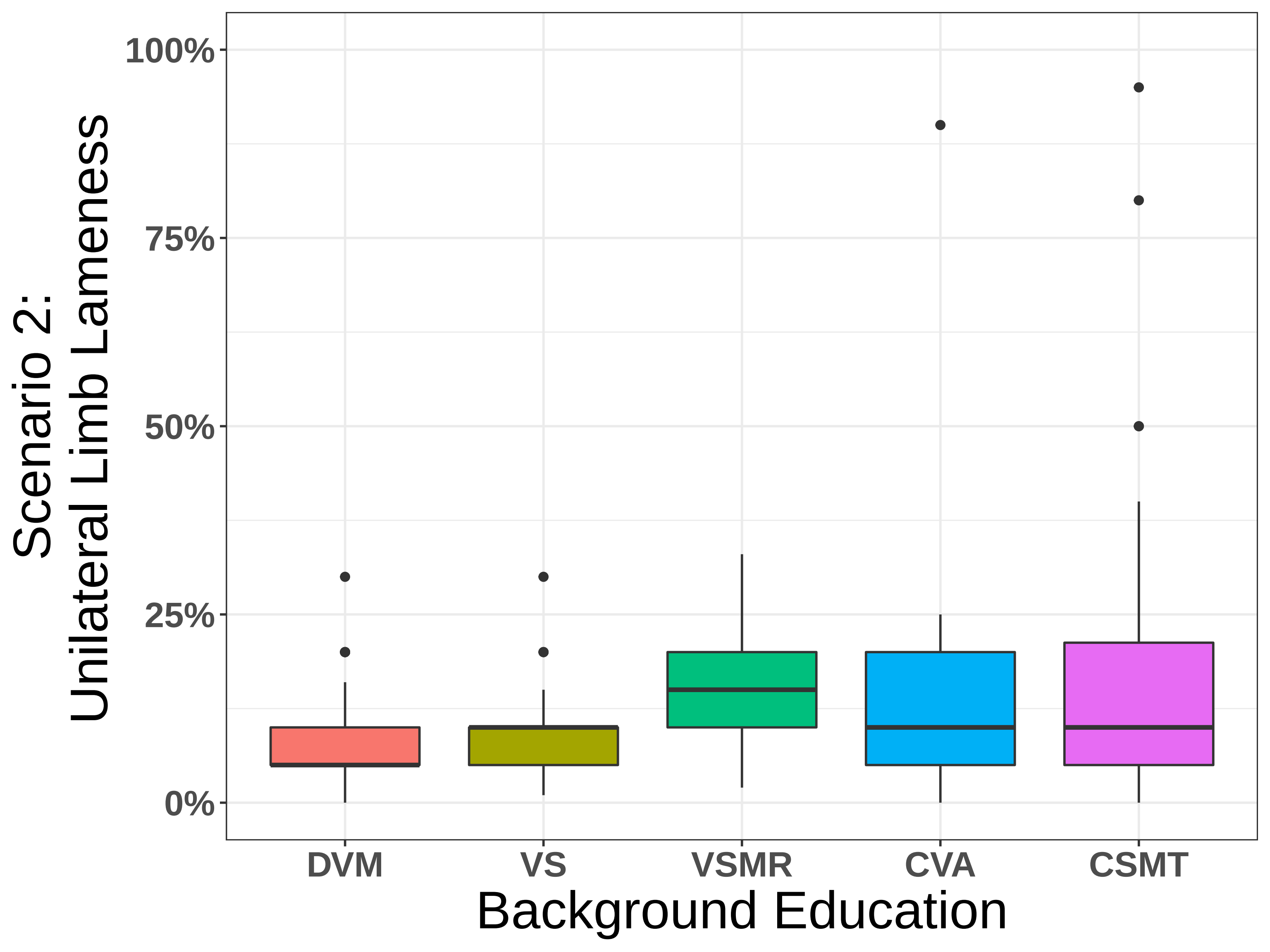

Scenario 2:
Unilateral Limb Lameness
100%
75%
50%
25%
0%
DVM
VS
VSMR
CVA
CSMT
Background Education

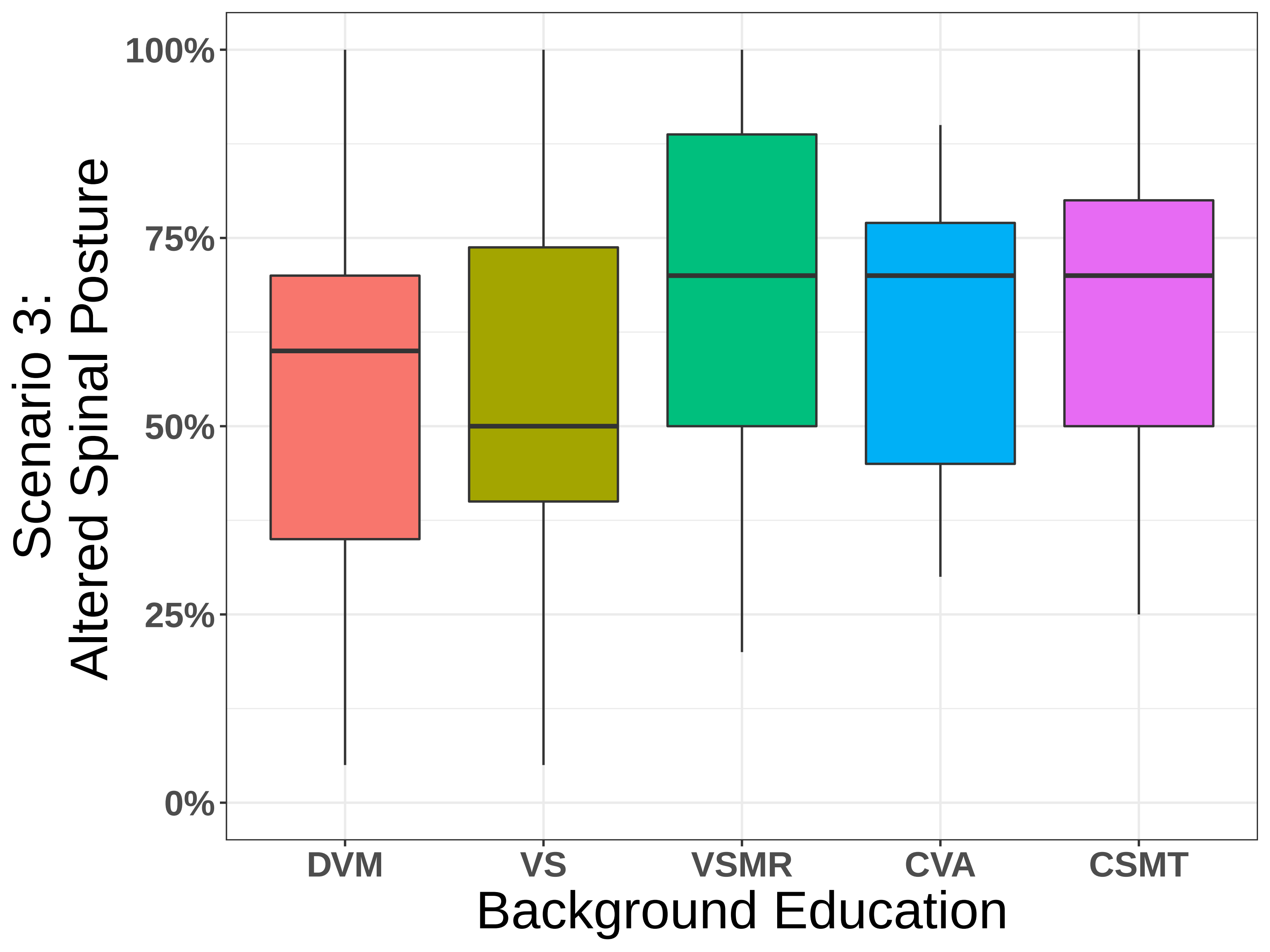
Scenario 3:
Altered Spinal Posture
100%
75%
50%
25%
0%
DVM
VS
VSMR
CVA
CSMT
Background Education

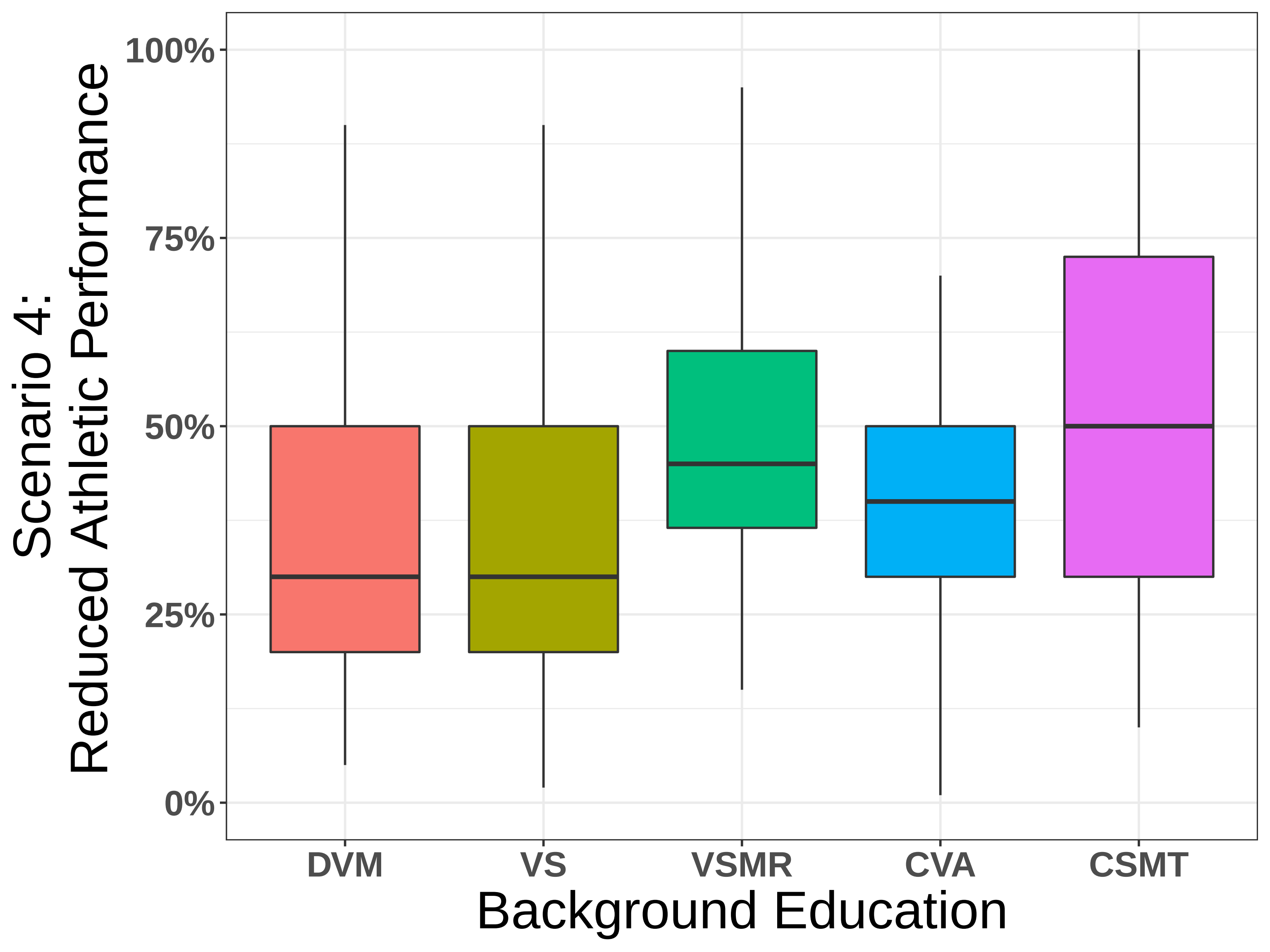

Scenario 4:
Reduced Athletic Performance
100%
75%
50%
25%
0%
DVM
VS
VSMR
CVA
CSMT
Background Education

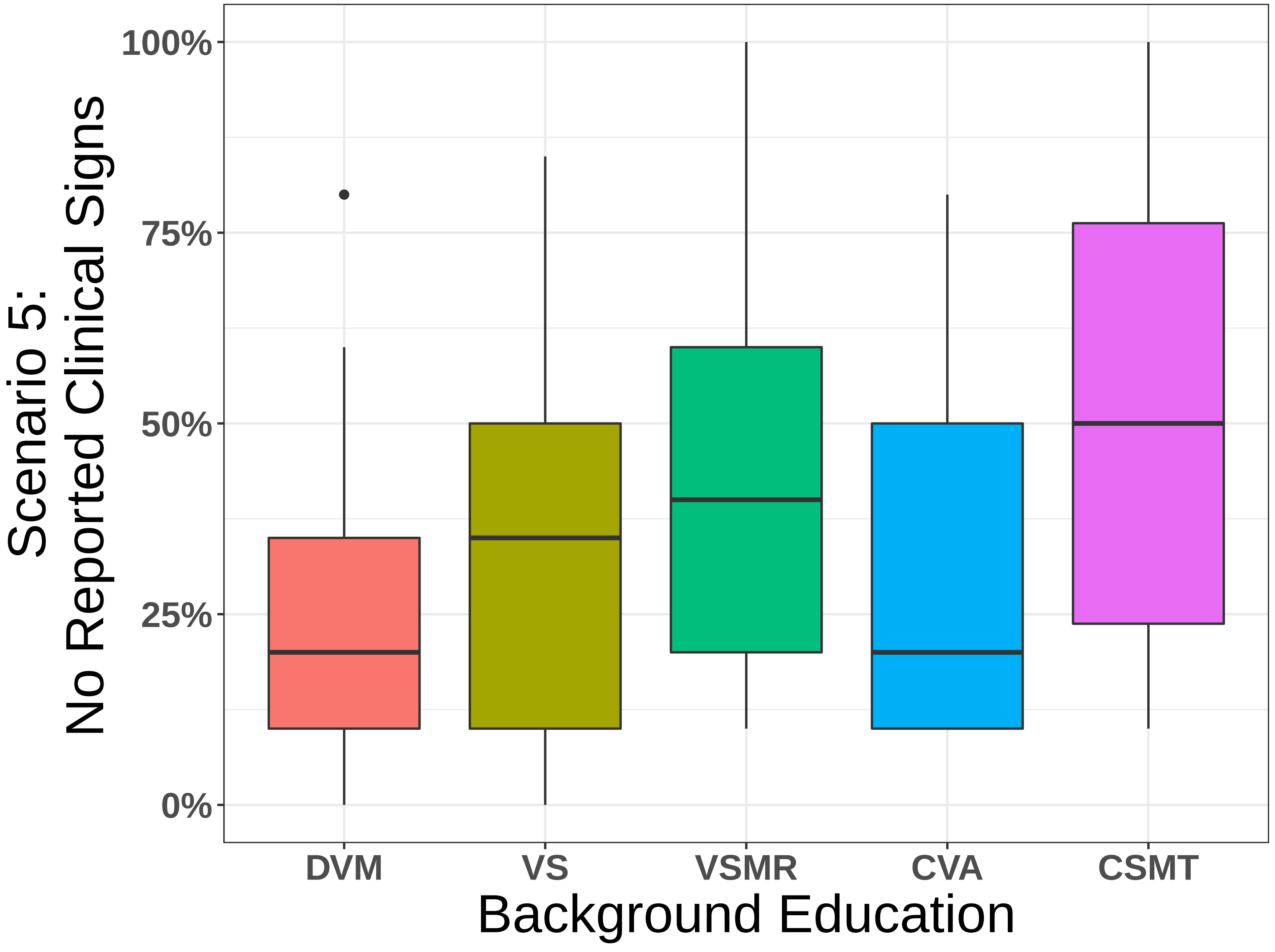

Scenario 5:
No Reported Clinical Signs
100%
75%
50%
25%
0%
DVM
VS
VSMR
CVA
CSMT
Background Education

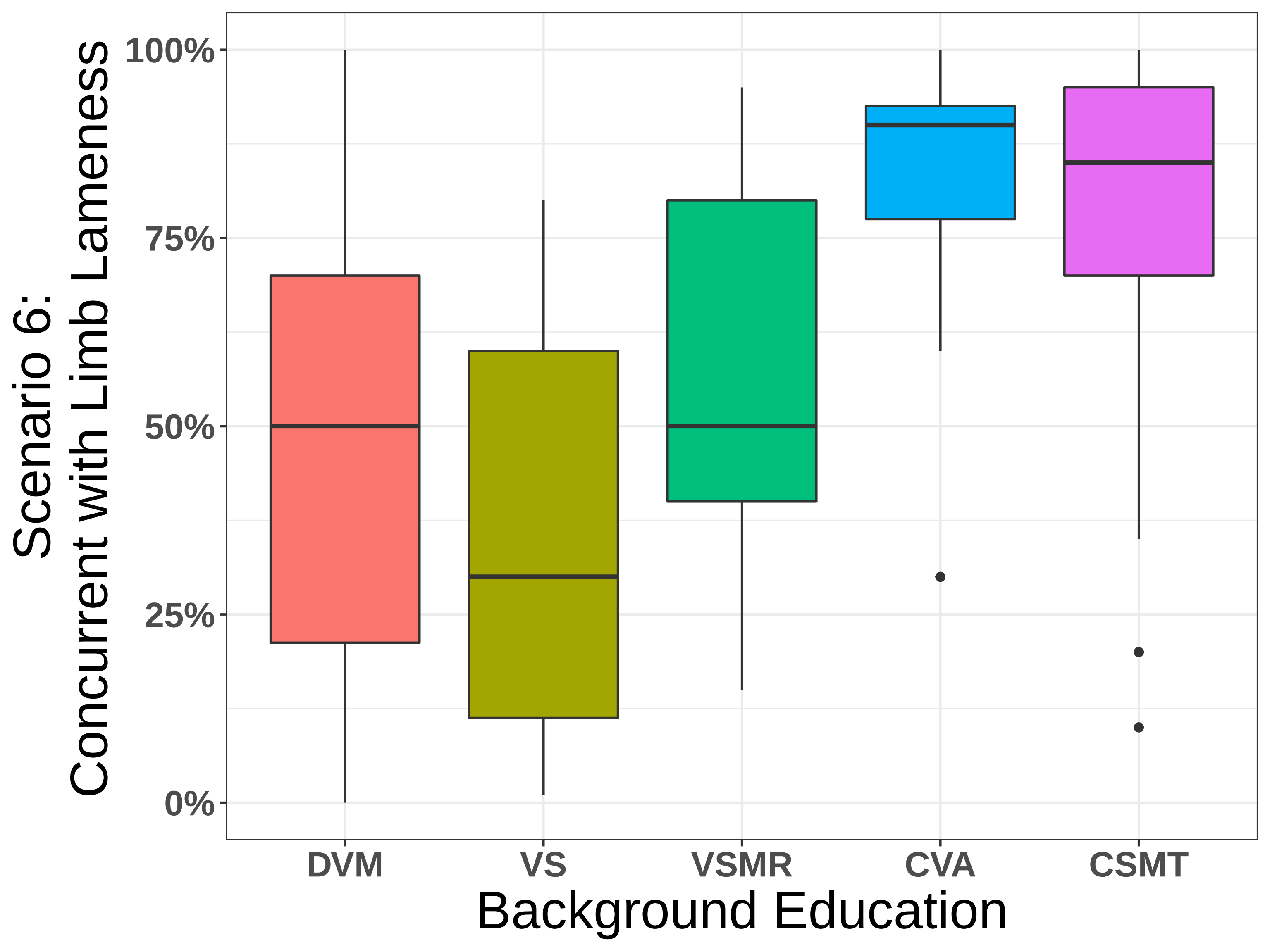
Scenario 6:
Concurrent with Limb Lameness
100%
75%
50%
25%
0%
DVM
VS
VSMR
CVA
CSMT
Background Education
