# Supplementary material for: Factors affecting veterinary perception of the prevalence of back pain and dysfunction in dogs and horses
Source: Front Vet Sci. 2026 Aug 17;13:1857696. doi: 10.3389/fvets.2026.1857696 (PMC13528434; doi:10.3389/fvets.2026.1857696)
Supplement: Supplementary file 3 [file Data_Sheet_3.PDF]

# Veterinary Perceptions of Back Pain

**The purpose of this survey is to understand veterinary perceptions of back pain – how often it occurs, and how it manifests. Collecting this data from veterinarians with a variety of practice backgrounds is the first step toward a larger goal of standardizing how back pain should be assessed and reported, and will help guide future research.**

**With gratitude,**

**David Lane DVM, ACVSMR**
**Kevin Haussler DVM, ACVSMR**

1. What degrees and certifications do you possess? Check all that apply.

- ☐ DVM or equivalent (e.g.: VMD, Dr.med.vet.)
- ☐ ACVSMR/ECVSMR board certification or residency
- ☐ ACVS/ECVS board certification or residency
- ☐ ACVIM/ECVIM (neurology) board certification or residency
- ☐ Rehabilitation certification (e.g.: CCRT, CCRP)
- ☐ Acupuncture certification
- ☐ Spinal manipulation therapy (chiropractic) certification (e.g.: CVSMT)
- ☐ Other (please specify)

2. Do you predominantly work in:

- ◯ Private Practice
- ◯ Academia
- ◯ Other (please specify)

3. Do you treat canines

- ◯ Yes
- ◯ No

## The following questions all pertain to canine education.

4. As part of your formal education in veterinary school, were you taught how to perform a detailed appendicular (limb) musculoskeletal examination?

- ◯ Yes
- ◯ No

5. As part of your formal education in veterinary school, were you taught how to perform a detailed axial (vertebral column) musculoskeletal examination?

- ◯ Yes
- ◯ No

6. Are there any comments you wish to make about your veterinary school education regarding musculoskeletal examinations? (optional)

[ ]

## The following questions all pertain to canine patients.

**Each scenario is asking about primary musculoskeletal pain or disfunction, excluding pain due to underlying systemic conditions such as tick borne, immune mediated, or neoplastic disease.**

**Please answer from the perspective of your practice caseload.**

**Axial = pertaining to the vertebral column**
**Appendicular = pertaining to the limbs**

7. For patients diagnosed with musculoskeletal pain, lameness, or dysfunction, how frequently is the primary lesion detected in each of the following locations:

Appendicular (fill in percentage)

[ ]

Axial (fill in percentage)

[ ]

Both axial and appendicular, and it is unclear whether the primary lesion is axial vs appendicular in origin (fill in percentage)

[ ]

Don't know the answer to the above question (type "X")

[ ]

8. What percentage of your canine patients diagnosed with primary appendicular lameness also have secondary or coincidental axial pain or dysfunction: (fill in percentage)

[ ]

9. For patients with a presenting complaint of unilateral limb lameness, how frequently is the primary lesion detected in each of the following locations:

Appendicular (fill in percentage)

Axial (fill in percentage)

Both axial and appendicular, and it is unclear whether the primary lesion is axial vs appendicular in origin (fill in percentage)

Don't know the answer to the above question (type "X")

10. For patients with a presenting complaint of altered spinal posture (lordosis, kyphosis, etc.), pain when turning or bending, and/or reluctance to move, how frequently is the primary lesion detected in the following locations:

Appendicular (fill in percentage)

Axial (fill in percentage)

Both axial and appendicular, and it is unclear whether the primary lesion is axial vs appendicular in origin (fill in percentage)

Don't know the answer to the above question (type "X")

11. For patients with a presenting complaint of non-specific reduced athletic performance but no lameness, (e.g.: reduced jump height, slower running speed), how frequently is the primary lesion detected in the following locations:

Appendicular (fill in percentage)

Axial (fill in percentage)

Both axial and appendicular, and it is unclear whether the primary lesion is axial vs appendicular in origin (fill in percentage)

Don't know the answer to the above question (type "X")

12. For patients with a presenting history of no known signs of musculoskeletal pain, dysfunction, or reduced athletic performance, how frequently is an abnormal finding detected during physical examination in the following locations:

Appendicular (fill in percentage)

Axial (fill in percentage)

Both axial and appendicular, and it is unclear whether the primary lesion is axial vs appendicular in origin (fill in percentage)

Don't know the answer to the above question (type "X")

## The following questions all pertain to canine patients

13. Which of the following most accurately describes how you typically assess the axial skeleton:

- ○ Global assessment (vertebral column as a whole)
- ○ Regional assessment (e.g.: cervical, thoracic, lumbar, pelvis)
- ○ Segmental assessment of each individual vertebra or motion unit
- ○ All of the above
- ○ Do not routinely assess the axial skeleton

## The following questions all pertain to canine patients

14. When examining each individual vertebra, which of the following are you assessing (check all that apply).

- ☐ Static or neutral resting position
- ☐ Passive joint range of motion
- ☐ Active joint range of motion
- ☐ Joint end feel
- ☐ Pain
- ☐ Other (please specify)

## The following questions all pertain to canine patients

15. When performing a routine examination of the axial skeleton do you assess the ribs?

- ◯ Yes
- ◯ No

## The following questions all pertain to canine patients

16. When examining each individual rib, which of the following are you assessing (check all that apply).

- ☐ Static or neutral resting position
- ☐ Passive joint range of motion
- ☐ Active joint range of motion
- ☐ Joint end feel
- ☐ Pain
- ☐ Other (please specify)

## The following questions all pertain to canine patients

17. When performing a routine examination of the axial skeleton do you assess the sacroiliac joints?

- ◯ Yes
- ◯ No

## The following questions all pertain to canine patients

18. When examining each sacroiliac joint, which of the following are you assessing (check all that apply).

- ☐ Static or neutral resting position
- ☐ Passive joint range of motion
- ☐ Active joint range of motion
- ☐ Joint end feel
- ☐ Pain
- ☐ Other (please specify)

## The following questions all pertain to canine patients

19. Which of the following are included in your examination of the paraspinal musculature (check all that apply):

- [ ] Muscle mass and/or development
- [ ] Muscle tone and texture
- [ ] Myalgia (muscle soreness)
- [ ] Myofascial trigger points
- [ ] Do not examine paraspinal musculature
- [ ] Other (please specify)

20. Do you treat equines?

- ( ) Yes
- ( ) No

## The following questions refer to equine education

21. As part of your formal education in veterinary school, were you taught how to perform a detailed appendicular (limb) musculoskeletal examination?

- ( ) Yes
- ( ) No

22. As part of your formal education in veterinary school, were you taught how to perform a detailed axial (vertebral column) musculoskeletal examination?

- ( ) Yes
- ( ) No

23. Are there any comments you wish to make about your veterinary school education regarding musculoskeletal examinations? (optional)

## The following questions refer to equine patients

**Each scenario is asking about primary musculoskeletal pain or disfunction, excluding pain due to underlying systemic conditions such as tick borne, immune mediated, or neoplastic disease.**

**Please answer from the perspective of your practice caseload.**

**Axial = pertaining to the vertebral column**

**Appendicular = pertaining to the limbs**

24. For patients diagnosed with musculoskeletal pain, lameness, or dysfunction, how frequently is the primary lesion detected in each of the following locations:

Appendicular (fill in percentage)

Axial (fill in percentage)

Both axial and appendicular, and it is unclear whether the primary lesion is axial vs appendicular in origin (fill in percentage)

Don't know the answer to the above question (type "X")

25. What percentage of your patients diagnosed with primary appendicular lameness, also have secondary or coincidental axial pain or dysfunction: (fill in percentage)

26. For patients with a presenting complaint of unilateral limb lameness, how frequently is the primary lesion detected in each of the following locations:

Appendicular (fill in percentage)

Axial (fill in percentage)

Both axial and appendicular, and it is unclear whether the primary lesion is axial vs appendicular in origin (fill in percentage)

Don't know the answer to the above question (type "X")

27. For patients with a presenting complaint of altered spinal posture (lordosis, kyphosis, etc.), bucking, and/or adverse behaviour with saddle placement, how frequently is the primary lesion detected in the following locations:

Appendicular (fill in percentage)

Axial (fill in percentage)

Both axial and appendicular, and it is unclear whether the primary lesion is axial vs appendicular in origin (fill in percentage)

Don't know the answer to the above question (type "X")

28. For patients with a presenting complaint of non-specific reduced athletic performance but no lameness, (e.g.: reduced jump height, slower running speed), how frequently is the primary lesion detected in the following locations:

Appendicular (fill in percentage)

[ ]

Axial (fill in percentage)

[ ]

Both axial and appendicular, and it is unclear whether the primary lesion is axial vs appendicular in origin (fill in percentage)

[ ]

Don't know the answer to the above question (type "X")

[ ]

29. For patients with a presenting history of no known signs of musculoskeletal pain, dysfunction, or reduced athletic performance, how frequently is an abnormal finding detected during physical examination in the following locations:

Appendicular (fill in percentage)

[ ]

Axial (fill in percentage)

[ ]

Both axial and appendicular, and it is unclear whether the primary lesion is axial vs appendicular in origin (fill in percentage)

[ ]

Don't know the answer to the above question (type "X")

[ ]

## The following questions all pertain to equine patients

30. Which of the following most accurately describes how you typically assess the axial skeleton:

- ○ Global assessment (vertebral column as a whole)
- ○ Regional assessment (e.g.: cervical, thoracic, lumbar, pelvis)
- ○ Segmental assessment of each individual vertebra or motion unit
- ○ All of the above
- ○ Do not routinely assess the axial skeleton


## The following questions all pertain to equine patients

31. When examining each individual vertebra, which of the following are you assessing (check all that apply).

- [ ] Static or neutral resting position
- [ ] Passive joint range of motion
- [ ] Active joint range of motion
- [ ] Joint end feel
- [ ] Pain
- [ ] Other (please specify)

## The following questions all pertain to equine patients

32. When performing a routine examination of the axial skeleton do you assess the ribs?

- ( ) Yes
- ( ) No

## The following questions all pertain to equine patients

33. When examining each individual rib, which of the following are you assessing (check all that apply).

- [ ] Static or neutral resting position
- [ ] Passive joint range of motion
- [ ] Active joint range of motion
- [ ] Joint end feel
- [ ] Pain
- [ ] Other (please specify)

## The following questions all pertain to equine patients

34. When performing a routine examination of the axial skeleton do you assess the sacroiliac joints?

- ( ) Yes
- ( ) No

## The following questions all pertain to equine patients

35. When examining each sacroiliac joint, which of the following are you assessing (check all that apply).

- ☐ Static or neutral resting position
- ☐ Passive joint range of motion
- ☐ Active joint range of motion
- ☐ Joint end feel
- ☐ Pain
- ☐ Other (please specify)

## The following questions all pertain to equine patients

36. Which of the following are included in your examination of the paraspinal musculature (check all that apply):

- ☐ Muscle mass and/or development
- ☐ Muscle tone and texture
- ☐ Myalgia (muscle soreness)
- ☐ Myofascial trigger points
- ☐ Do not examine paraspinal musculature
- ☐ Other (please specify)
